# Bidirectional nuclear polarization through electric dipole spin resonance enabled by spin-orbit interaction in a single hole planar quantum dot device

**DOI:** 10.1038/s41534-025-01075-0

**Published:** 2025-08-07

**Authors:** Sergei Studenikin, Jordan Ducatel, Olivia Ellis, Marek Korkusinski, Alex Bogan, Piotr Zawadzki, D. Guy Austing, Andrew Sachrajda

**Affiliations:** 1 Emerging Technologies Division, National Research Council of Canada, Ottawa, ON Canada; 2 Department of Physics, University of Ottawa, Ottawa, ON Canada

**Keywords:** Physics, Condensed-matter physics, Electronics, photonics and device physics, Quantum physics

## Abstract

Spin exchange between confined holes and nuclei has been demonstrated for zero-dimensional quantum dots by optical techniques but has not been observed for gated planar structures. Here, enabled by strong spin-orbit interaction, and under microwave (MW) illumination, we report hyperfine interaction and dynamic polarization of the nuclei *with confined heavy-holes* in a GaAs/AlGaAs double quantum dot device. Distinct signatures of the resultant hyperfine field on the electron dipole spin resonance (EDSR) signal include: hysteresis on sweeping the magnetic (B-) field up and down with characteristics that are strongly dependent on both MW power and B-field sweep rate; free bidirectional dragging of the EDSR condition; stable locking on resonance on a timescale of hours; slow temporal change as the hyperfine field decays (T_1_ nuclear decay time ~ 100 s); and oscillations in time commensurate with Larmor precession of the ^75^As nuclei. We attain pumped nuclear (Overhauser) fields ~ 25 mT (~20% nuclear polarization).

## Introduction

Hole spins in semiconductor quantum dots (QDs) have attracted a growing interest for qubits due to the following desirable properties: large spin-orbit interaction (SOI); anisotropic g-factors; and, compared to electron spin, a greatly diminished (order of magnitude weaker) and anisotropic hyperfine coupling to the nuclei of the crystal lattice- see ref. ^1^ for a comprehensive review. The weaker hyperfine coupling for hole spin stems from the p-orbital character of the valence bands that essentially eliminates the contact interaction component dominant for electron spins, leaving the smaller dipole-dipole interaction component^2–7^. The reduced strength of the coupling promotes long hole spin relaxation and coherence times^2,3,8–10^. The impact of strong hyperfine coupling for electron spin leading to a variety of phenomena related to dynamic nuclear polarization (DNP) through so-called flip-flop processes has a long history encompassing work on two-dimensional electron gases (2DEGs) at odd filling factor under MW illumination^11,12^; spin-unpolarized and spin-polarized domains at ν = 2/3 filling in the fractional quantum Hall regime^5,13^; scattering between edge channels in quantum point contacts (QPCs) and quantum Hall devices^5,14,15^; quantum Hall effect breakdown at high current^16^; and likewise for confined electrons in double quantum dots (DQDs) in the spin-blockade regime^17,18^, and in optically pumped self-assembled QDs^19,20^. Regarding the impact of hyperfine coupling for hole spin, the theoretical prediction that the strength of the coupling is ~10% that for electron spin^2,21^ was corroborated optically with InGaAs self-assembled QDs^7,22,23^. On the other hand, clear signs of hyperfine coupling for hole spin in two-dimensional-hole-gas (2DHG) based electronic devices such as QPCs or planar QDs are yet to be reported: see the null results for resistively detected nuclear magnetic resonance in studies featuring p-type GaAs QPCs in refs. ^24,25^.

Here, we demonstrate that through the combination of strong SOI and MW illumination confined heavy-holes (henceforth holes for brevity) can interact with the (10^5^–10^6^) nuclei of the host atoms of a gated planar GaAs/AlGaAs QD structure^1,26–29^. Hallmarks of hyperfine interaction and dynamic polarization of the nuclei commonly observed with electrons are reported for holes by EDSR, namely signal hysteresis, asymmetric line shape, slow change in signal position, and for pulsed MWs, oscillations at a Larmor frequency for nuclear species present. We present a model (Section II of the Supplementary Note) whereby the strong SOI in conjunction with the hyperfine interaction leads to different nuclear spin quantization axes in the hole-ground-state-manifold and in the hole-first-excited-state-manifold which facilitates nuclear spin pumping. The manifolds here are composed of states of the hole coupled with the nuclear spin system. A distinguishing feature of our work is bidirectionality whereby a nuclear field with positive or negative polarity builds up to compensate the applied B-field allowing the EDSR condition to be satisfied. The resonance can be dragged equally to higher or lower B-field away from the (pure) EDSR condition in the absence of nuclear polarization which leads to identical but inverted (“mirror image”) EDSR signals with respect to the B-field sweep direction. The bidirectional dragging of the EDSR signal featuring a hole spin-nuclear spin feedback process mediated by SOI that we observe is distinct from the unidirectional dragging featuring an electron spin-nuclear spin feedback process in refs. ^30–32^ for GaAs/AlGaAs DQD devices, confining two electrons and in the Pauli spin-blockade regime, for which SOI plays no role. In ref. ^30^, excited by a radio-frequency B-field generated on chip, electron spin resonance is dragged in one direction only as the B-field (or excitation frequency) is swept, i.e., DNP is unipolar. The work in ref. ^31^ is similar other than EDSR at MW frequency is employed with a DQD device that incorporates a micro-magnet. More generally, in experiments with DQDs that in the absence of a resonant excitation polarize the nuclei by sweeping the B-field or dot detuning in the vicinity of an avoided crossing between two-electron singlet and triplet states (S-T^+^ *or* S-T^-^) DNP is predominantly in one direction only^33–35^. Bidirectional polarization has been reported for a scheme that alternately switches between opposing S-T^+^ and S-T^-^ pumping points^36,37^- see also the related scheme in ref. ^38^ where two opposing pumping points are brought into coincidence. Furthermore, while it has been demonstrated that nuclear polarization of opposite sign can be built up by sweeping in opposite directions through the S-T^+^ avoided crossing the initially prepared electronic states have different spin^39^.

## Results

### Double quantum dot device and bidirectional dragging of EDSR signal in single-hole regime

**Fig. 1:**
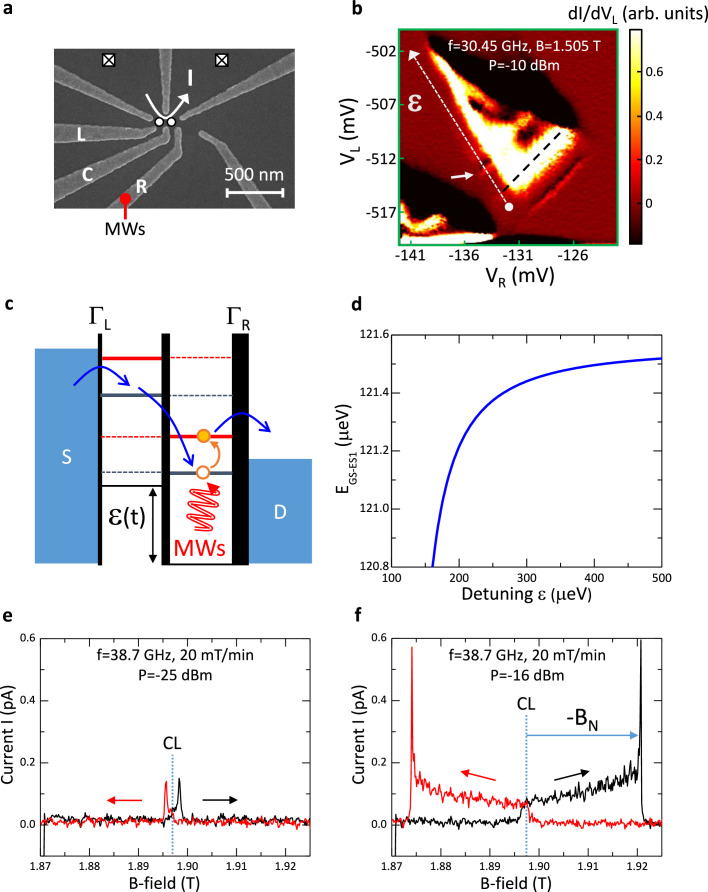
Double quantum dot device and bidirectional dragging of EDSR signal in single-hole regime. The device layout is shown in (**a**) (the global accumulation gate is absent in the image). The crossed boxes denote Ohmic contacts. Single-hole high-bias (V_SD_ = 0.5 mV) transport triangle at B = 1.505 T with MWs applied at f = 30.45 GHz and P = −10 dBm is shown in (**b**). Here dI/dV_L_ is plotted as a function of the voltages V_L_ and V_R_ on the gates L and R respectively. Along the white dashed arrow, the gate voltages are changed in such a way that the energy level detuning ε is swept in a positive direction away from the base of the transport triangle (black dashed line corresponding to zero detuning), and the trajectory cuts through the EDSR line feature indicated by the white arrow (the corresponding EDSR line feature at negative detuning is also visible below the transport triangle)^29^- see also Section I of the Supplementary Note. Cartoon indicating the alignment of quantum molecular levels at positive detuning when the system is in an energy blockaded condition is depicted in (**c**). MW modulation can excite a trapped spin-down hole on the right QD into an excited state whereby the hole exits to the drain (D). **d** The calculated energy gap between the ground state and first excited state E_GS-ES1_ as a function of detuning ε for parameters: spin-conserving tunneling matrix element t_N_ = 8 μeV, spin-flipping tunneling matrix element t_F_ = 6 μeV, B = 1.5 T, bulk-effective g-factor g*=1.4. See Supplementary Fig. 1 for calculated eigenenergies of the lowest four energy levels^1,29^. **e**, **f** show, respectively, the EDSR signal at low power (P = −25 dBm) and higher power (P = −16 dBm). I is measured as a function of B with f = 38.7 GHz. The B-field is swept up (black trace) and down (red trace) at rate 20 mT/min. The vertical dashed line CL marks the effective B-field position of the pure EDSR peak (B = 1.897 T). At a given MW power, the position of CL empirically is taken to be the mid-point between the EDSR signal maxima for the up- and down- sweep traces.

A micrograph of the lateral DQD device investigated is shown in Fig. 1a. The device is fabricated from an undoped GaAs/Al_0.5_Ga_0.5_As heterostructure^1,29,40^ (see Method section). Voltages applied to the left (L) and right (R) plunger gates tune the hole energy levels in the QDs, while the voltage on the center (C) gate regulates interdot tunneling. Current (I) flows through the DQD in the direction indicated in response to a *positive* source-drain bias V_SD_. MW modulation is applied to gate R and a B-field is applied perpendicular to the 2DHG. As demonstrated elsewhere^1,29^, we can reach the single-hole regime. As exemplified by the single-hole high-bias (V_SD_ = 0.5 mV) transport triangle at B = 1.505 T in Fig. 1b with MWs applied at frequency f = 30.45 GHz and (nominal) power P = −10 dBm an EDSR line feature (white arrow) at positive dot detuning is induced just outside the transport triangle^29^- see also Section I of the Supplementary Note. The energy band cartoon in Fig. 1c illustrates how the EDSR arises. Because of the strong SOI, the single-hole quantum molecular levels are superpostions in both spatial occupation and spin. At sufficiently large positive dot detuning ε relevant to our discussion, MW modulation can excite a hole that is spin-down and trapped on the right QD from the ground state into an excited state where the hole has both spin-up and spin-down components and is more delocalized spatially (Supplementary Fig. 1). The excited hole then exits to the drain. As illustrated in the calculation for B = 1.5 T in Fig. 1d the energy gap between the ground state (GS) and first excited state (ES1) E_GS-ES1_ that the MWs must bridge depends not only on the B-field but also the detuning over a range of a few hundreds of micro-electron volts. The energy gap for EDSR can be characterized by an effective g-factor g_eff_(ε) = hf/μ_B_B that is *electrically tunable*^1,29^- here h (μ_B_) is Planck’s constant (the Bohr magneton).

When we perform EDSR with continuous MW modulation we find in general that the EDSR signal does not have a simple line shape. Rather, over a wide range of MW powers the EDSR signal displays a complex (asymmetric) line shape and pronounced hysteresis on sweeping B up and down. Figure 1e, f, give examples for a B-field sweep rate of 20 mT/min. At low MW power (P = −25 dBm: Fig. 1e), the EDSR signal is almost symmetric and the up- and down-sweep signals are separated by ~2–3 mT. At higher MW power (P = −16 dBm: Fig. 1f), the EDSR signal in either sweep direction becomes trapezoidal and extends over a ~25 mT range. Distinctively, the up-sweep and down-sweep signals are mirror images of each other on reflection around a center line (CL) at a B-field where the pure EDSR (sharp) peak is expected as set by f and g_eff_. Supplementary Fig. 2 shows the full data set. We ascribe our observations to a bidirectional dragging effect related to the dynamic polarization of the nuclei with holes. CL marks the B-field position of the pure EDSR peak in the absence of nuclear polarization. Relative to CL, from the position of the EDSR signal maximum in each direction, the maximum built-up nuclear field B_N_ in e (f) is estimated to be ~1 mT ( ~23 mT) in magnitude. B_N_ has a negative (positive) sign on the up-sweep (down-sweep). Note that even at P = −34 dBm in Supplementary Fig. 2, the smallest MW power for which an EDSR signal is still discerned, B_N_ is small but finite ( ~0.5 mT). Supplementary Fig. 3 shows a second data set for a 40 mT/min sweep rate with MW power up to P = + 2 dBm illustrating the generality of our observations: the maximum B_N_ for the P = −4 dBm traces is ~20 mT (at higher MW power the maximum nuclear field achieved is diminished). We outline our model for the bidirectional dragging and nuclear polarization effect in the Methods section which we develop further in Section II of the Supplementary Note. The rising edge of the EDSR signal in the traces shown in Fig. 1e, f (see also Supplementary Figs. 2, 3 and 4), reflects the onset of nuclear polarization and the position is within a few mT of the expected pure EDSR peak. The EDSR signal then generally grows steadily as the nuclear polarization builds up. The growth in the EDSR results from a detailed competition between nuclear spin pumping, nuclear spin relaxation, and the B-field sweep rate. For moderate MW powers (and slow sweeping of the B-field), the signal can be sustained over a range up to ~25 mT. The trailing edge of the EDSR signal, often accompanied by a pronounced increase in the current before an abrupt drop in current, marks the point where the nuclear polarization can no longer grow to compensate the applied B-field and the EDSR condition is lost, i.e., the depolarization rate exceeds the polarization rate (Section II of the Supplementary Note).

### Characteristics of EDSR dragging and locking

**Fig. 2:**
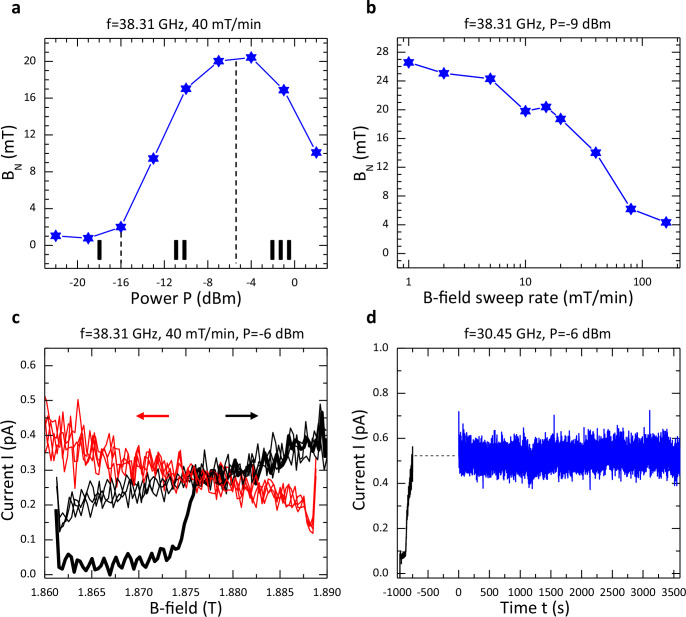
Characteristics of EDSR dragging and locking. Maximum built-up nuclear field B_N_ as a function of MW power P on sweeping the B-field up and down at rate 40 mT/min (**a**), and as a function of B-field sweep rate on sweeping the B-field up and down at *P* = −9 dBm (**b**). In (**a**, **b**) the MW frequency is 38.31 GHz. See Supplementary Figs. 3 and 4 for data sets. See Supplementary Fig. 5 for a compilation of maximum B_N_ for an extensive set of measurements where both the MW power and the B-field sweep rate are varied. First example of locking (**c**). I as a function of B-field with MWs applied at f = 38.31 GHz and *P* = −6 dBm. The B-field sweep rate is 40 mT/min. The B-field is repeatedly swept up (black traces) and down (red traces) between 1.86 and 1.89 T. At the end of each sweep, the MWs remain on for 60 s (during which I is not recorded), and then the MWs are turned off for 10 s (during which there is no EDSR signal) before the commencement of the following sweep. Note that at the start of the first up sweep (bold black trace) the current is zero (off-resonance initial condition) and any prior nuclear field has fully relaxed. The onset of finite current at ~1.875 T indicates the initial resonance condition has been attained. For this experiment, V_SD_ = 0.5 mV and ε~310 μeV. See Supplementary Fig. 7 for data plotted as a function of time. Second example of locking (**d**). I as a function of time t with MWs applied at f = 30.45 GHz and P = −6 dBm. From t = −960 to −750 s the B-field is swept up from 1.495 T (off-resonance initial condition) to 1.53 T (on-resonance condition) at 10 mT/min (black trace). Thereafter the B-field is held constant at 1.53 T. From t = −750 to 0 s I was not recorded. Starting at t = 0 s I was recorded for a duration of 1 h (blue trace) and the current is locked (on-resonance) to a value ~ 0.5 pA. For this experiment, V_SD_ = 0.5 mV and ε~300 μeV.

We now determine the maximum nuclear field that can be attained on sweeping the B-field and its dependence on MW power and B-field sweep rate. Figure 2a shows the maximum value of B_N_ as a function of MW power with a fixed B-field sweep rate of 40 mT/min and for f = 38.31 GHz (Supplementary Fig. 3). Three regimes marked I, II, III are identified. In regime I, B_N_ is small (~1mT) and has a weak dependence on power up to P ~ −16 dBm. In regime II, B_N_ grows rapidly at first before reaching a limit of ~20 mT at P ~ −4 dBm. In regime III, B_N_ decreases. In our model, the three regimes mark the situation when the amplitude in the oscillation in the energy gap E_GS-ES1_ in the MW excitation process is respectively smaller than, comparable to, and larger than the half-width of a spectral function describing the amplitude of possible transitions between the hole-ground-state-manifold and the hole-first-excited-state-manifold (see Methods section). Hereafter we focus on measurements in regimes I and II. Figure 2b shows the maximum value of B_N_ as a function of B-field sweep with a fixed MW power of -9 dBm (regime II) and for f = 38.31 GHz (Supplementary Fig. 4). Sweeping the B-field slowly allows the maximum possible nuclear field to build up whereas for high sweep rates the EDSR condition is engaged for insufficient time to strongly pump the nuclear spin. Supplementary Fig. 5 shows the maximum B_N_ for an expanded set of measurements illustrating the robustness of our observations and highlights other trends not directly evident in Fig. 2a, b. The maximum value of B_N_ determined from the data shown in Fig. 2b is ~26 mT. Taking the hyperfine parameters for the valence-band in ref. ^41^ and accounting for the abundances of the ^69^Ga, ^71^Ga, and ^75^As isotopes (all with nuclear spin I = 3/2), full nuclear polarization translates to a nuclear field of ~115 mT for heavy-holes^4^ (see Methods section for discussion). For comparison 100% polarization translates to a nuclear field of ~5 T for electrons in GaAs. Hence we determine a maximum nuclear polarization of 20–25% has been achieved with holes by EDSR dragging for a slow (1 mT/min) sweep rate in Fig. 2b. This value compares to reported nuclear polarizations of ~40% to ~80% (and recently even 95% or higher) achieved electrically or optically with various QD structures through flip-flop exchange between electron spin and nuclear spin^33,42–45^.

Figure 2c, d illustrate two manifestations of locking, namely we can freely drag in a bidirectional manner the EDSR condition, or maintain a fixed on-resonance condition for a timescale of at least an hour. The first example of locking is shown in Fig. 2c (see Methods section for full description). The B-field is repeatedly swept up (black traces) and down (red traces) at 40 mT/min with MWs applied at f = 38.31 GHz and P = -6 dBm. Other than at the start of the first up-sweep (bold black trace) when the initial condition is off-resonance, once the onset of finite current at ~1.875 T has been achieved, the on-resonance condition can be freely dragged in the 1.86 to 1.89 T window of operation. The data in Fig. 2c is replotted as a function of time in Supplementary Fig. 7 along with the reconstructed nuclear field that dynamically changes to maintain EDSR as the B-field is swept. In contrast to the bidirectional dragging effect through a hole-nuclear feedback mechanism observed here, only unidirectional dragging involving the locking of electron spins into resonance through an electron-nuclear feedback mechanism has been previously reported^30^: see also ref. ^32^. The second example of locking is shown in Fig. 2d. The B-field is swept up from an off-resonance initial condition to and on-resonance condition (black trace) with MWs applied at f = 30.45 GHz and *P* = −6 dBm. Thereafter, the B-field is held constant at 1.53 T, and apart from a short ( ~750 s) period of time at the start of the experiment when the current was not recorded, the EDSR current is maintained at a constant value of ~0.5 pA for a duration of one hour (blue trace), i.e., the Overhauser field locks essentially indefinitely when the B-field is stopped on-resonance. Stable locking of electron spins for up to a couple of minutes was reported in ref. ^30^. We note that long duration stable albeit oscillatory nuclear spin pumping phenomena with electrons have been reported^17,46^.

### Pump-probe measurement of nuclear spin relaxation time

**Fig. 3:**
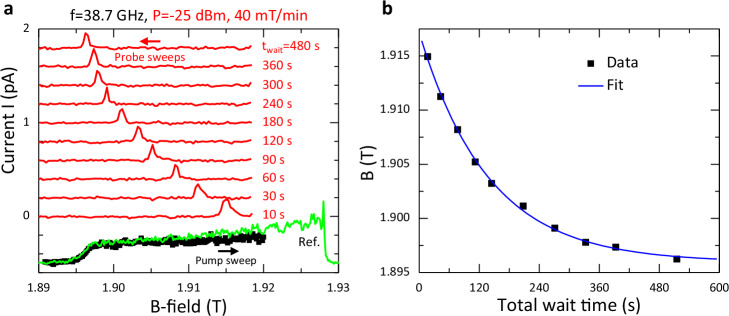
Pump-probe measurement of nuclear spin relaxation time. In the experiment the nuclear spin is pumped at high MW power (P = −9 dBm) on a slow up-sweep (sweep rate 20 mT/min) and, after a varying wait time t_wait_, with the MWs turned off, the nuclear spin is probed at low MW power (P = −25 dBm) on a fast down-sweep (sweep rate 40 mT/min). Down-sweep (red) traces of I versus B-field that probe the nuclear spin relaxation for different values of t_wait_ between 10 and 480 s are vertically offset relative to the t_wait_ = 10 s trace for clarity (**a**). *One* of the (nominally identical) up-sweep (black) traces that pumps the nuclear spin is also shown (offset down for clarity). Prior to each up-sweep, the MWs are turned-off for 120 s. The up-sweep starts at 1.89 T off-resonance and ends at 1.92 T *on-resonance*. Additionally, a reference up-sweep (green) trace, taken immediately before the experiment, also for P = −9 dBm and 20 mT/min sweep rate, showing the full EDSR signal is superimposed on the black trace. Here the EDSR signal ends near B ~ 1.93 T. For this experiment, f = 38.7 GHz, V_SD_ = 1 mV and ε~490 μeV. From the exponential decay of the B-field position of the down-sweep peak with t_wait_ in **a**, a T_1_ relaxation time T_N_ of 139 s is determined from a fit of the data points (**b**). Note that the *total* wait time corresponds to t_wait_ plus the time taken from the start of the probe sweep to reach the down-sweep peak.

We next perform a pump-probe measurement to determine the Overhauser field relaxation time. Figure 3a shows a sequence of fast (40 mT/min) down-sweep (red) traces, taken at low MW power (*P* = −25 dBm) that probe the residual nuclear polarization after a varying wait time t_wait_ with the MWs turned off before which the nuclear spin had been pumped on-resonance at high MW power (*P* = −9 dBm) on a slow (20 mT/min) up-sweep, representative trace coloured black. Figure 3b plots the B-field position of the narrow down-sweep peak as a function of *total* wait time (t_wait_ plus the time taken from the start of the probe sweep to reach the down-sweep peak). For the experimental conditions here, from the exponential decay, the T_1_ relaxation time is 139 s (see Methods section for discussion). This slow decay timescale for the Overhauser field in the presence of holes compares with reported decay timescales for the Overhauser field in the presence of electrons of seconds to a few hundred seconds also for pump-probe measurements with DQDs^30,31,33–35^.

### Microwave burst experiment and periodic oscillation of EDSR signal in time at Larmor frequency of ^75^As

**Fig. 4:**
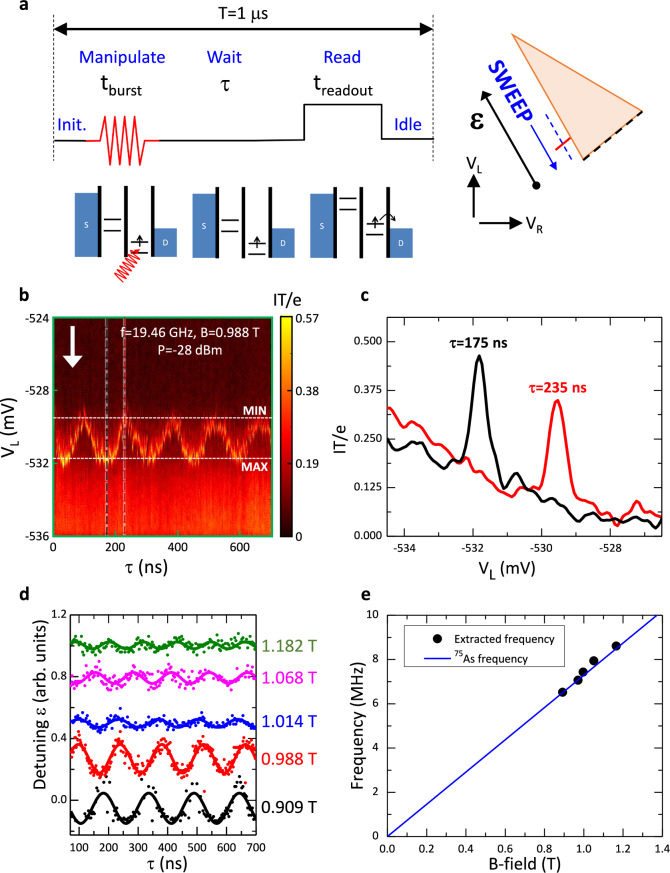
Microwave burst experiment and periodic oscillation of EDSR signal in time at Larmor frequency of ^75^As. The protocol implemented is depicted in (**a**) and fully explained in the Methods section. (**b**) shows a colour plot of the current measured as a function of swept V_L_ (white arrows marks scan direction) and stepped wait time τ at B = 0.988 T with the MW frequency and power respectively set to f = 19.46 GHz and P = −28 dBm. Note that the current is given in terms of IT/e (with period T = 1 μs), i.e., essentially the probability that a spin-up hole is read out although we have not subtracted the non-resonant background current (0.1 pA corresponds to probability 0.625). For this experiment, V_SD_ = 0.5 mV, t_burst _= t_readout_ = 100 ns, and the initialization (Init.) time is also 100 ns. The position in detuning of the EDSR signal maximum periodically oscillates with τ between maximal dragging (V_L_ ~ −532 mV corresponding to ε~250 μeV) and minimal dragging (V_L_ ~ −529.5 mV corresponding to ε~430 μeV): see dotted horizontal lines labelled MAX and MIN. **c** shows examples of the EDSR signal at maximal dragging (τ = 175 ns: black trace), and at minimal dragging (τ = 235 ns: red trace). The traces correspond to the vertical sections (dashed lines) through the plot in (**b**). (**d**) provides a compilation of the EDSR signal position in detuning (arb. units) as a function of τ extracted from five data sets with B = 0.909 T (black: f = 17.80 GHz, P = −10 dBm), 0.988 T [red: data shown in (**b**)], 1.014 T (blue: f = 20.01 GHz, P = −15 dBm), 1.068 T (purple: f = 21.25 GHz, P = −10 dBm), and 1.182 T (green: f = 23.58 GHz, P = −10 dBm). Other than the B-field, MW power and MW frequency, the experimental conditions are identical. The traces are uniformly offset from each for clarity. See Supplementary Fig. 8 for 0.909 and 1.068 T data sets. (**e**) shows the frequency of the oscillations from the data sets in (**d**) as a function of applied B-field. The extracted frequencies lie on a line that extrapolates to the origin. The frequencies closely match the expected Larmor frequencies for ^75^As: see blue line with slope 7.29 MHz/T.

Nuclear magnetic resonance with a continuous AC magnetic field applied perpendicular to the DC magnetic field is a definitive hallmark of nuclear spin^5,7,17^. In the absence of a nearby coil or on-chip stripline, we enlist the SOI to periodically generate a Knight field, equivalent to an effective AC magnet field, by a double-pulse technique, and observe oscillations commensurate with Larmor precession of the ^75^As nuclei: see also the similar technique in ref. ^47^. The experimental protocol is depicted in Fig. 4a (see Methods section for description). Figure 4b shows a plot of the current converted to IT/e (essentially the probability that a spin-up hole is read out where T = 1 μs is the period of the pulse sequence and e is the elementary charge) on sweeping the detuning (equivalent to V_L_) and stepping the wait time τ at B = 0.988 T with the MWs set to f = 19.46 GHz and P = −28 dBm. The key observation is that the EDSR signal maximum position in detuning periodically oscillates with τ between V_L_ ~ −532 mV (ε~250 μeV), where a nuclear field has accumulated and there is maximum dragging of the EDSR signal (MAX), and V_L_ ~ -529.5 mV (ε~430 μeV), where the nuclear field is essentially zero and there is minimal dragging of the EDSR signal (MIN). Figure 4c shows examples of the EDSR signal at maximal dragging (τ = 175 ns: black trace) and at minimal dragging (τ = 235 ns: red trace). Figure 4d shows the oscillations in the EDSR position in detuning as a function of wait time for five values of B-field between 0.909 and 1.182 T (Supplementary Fig. 8). The period of oscillation is B-field dependent. The extracted frequencies of the oscillations are plotted as a function of B-field in Fig. 4e. The data points lie on a line that extrapolates to the origin and the slope is very close to the Larmor frequency expected for ^75^As (blue line: 7.29 MHz/T). We have also examined the MW burst time (t_burst_) dependence with τ set to maximal and minimal dragging conditions (Supplementary Fig. 9).

## Discussion

Facilitated by SOI which activates spin-exchange we have demonstrated that the EDSR signal is broadened due to the coupling of the hole-spin and the spins of the nuclei of lattice atoms, i.e., the EDSR frequency depends on the nuclear polarization. In turn, the hole-spin acts as a local magnetic field for the nuclei. We have established the capability, hitherto lacking, to access nuclear spin electrically in planar QD structures confining holes which is potentially useful for quantum memory functionality^9,41,48^. Uniquely, we can pump the nuclear spin bidirectionally at a single pumping point dependent on the direction the EDSR condition is approached, in our case on sweeping the B-field, and the pumping action is the same in either direction. Nuclear state narrowing to counter nuclear randomness by locking the hyperfine field to a given value is attractive^4,7,30,39,49–51^. The native bidirectional pumping we have achieved may give enhanced performance over schemes that rely on competition between unidirectional pumping and nuclear spin relaxation mechanisms^49^- see refs. ^39,51,52^ for bidirectional pumping schemes featuring spin-exchange between electrons and nuclei. Bidirectional pumping and locking of the hyperfine field featuring spin-exchange between holes and nuclei via coherent dark-state manipulation has been reported for InAs self-assembled QDs^50^. Our observations are applicable to other group III-V materials, where non-zero nuclear spin isotopes are unavoidable, and potentially to group IV materials grown with natural rather than isotopically purified sources. Finally, our work can shed further light on the microscopic details of the hyperfine interaction with holes, for example, refs. ^41,53^ discuss the important contribution of d-orbitals.

## Methods

### Device and measurement details

Ti/Au surface gates are employed to create a DQD potential. A 2DHG is formed by a global accumulation gate on a dielectric layer deposited above the heterostructure^1,29,40^. A negative voltage on the accumulation gate, attracts holes from the contacts into the conducting channel. The DQD device is measured in a dilution refrigerator and the nominal lattice (effective hole) temperature is ~60 mK ( ~100 mK)^54^. Unless otherwise stated, the MWs are continuously applied. An Anritsu MW signal generator Model 69377B is used and the nominal MW power *at source* P is set between −34 and +2 dBm for the measurements described. In addition, the MW signal is attenuated by −20 dB at the 1 K stage and there is additional frequency-dependent loss in the coaxial lines. We stress that any MW power quoted is the nominal MW power set at the source and no correction has been made for the added attenuation at the 1 K stage or any frequency-dependent losses in the coaxial lines. For the MW burst measurements (Fig. 4) we employ a Keysight E8267D PSG vector signal generator. The burst time and amplitude are controlled by a Tektronix AWG 70002 A arbitrary waveform generator. The coupling of the left (right) QD to the source (drain) contact is Γ_L_ ~ 7 GHz (Γ_R_ ~ 120 MHz) at 0 T^54^ (Fig. 1c). The asymmetric coupling promotes rapid reoccupation of a single hole from the source (S) into the ground state (GS) when the DQD is empty, and ensures the trapped hole cannot easily exit to the drain (D) when the system is energy blockaded unless MWs can bridge the energy gap to the first excited state (ES1). See refs. ^1,29,55^ for detailed discussion of the model employed to calculate the eiegenenergies of the four lowest single-hole levels as a function of detuning in Supplementary Fig. 1.

As well as B-field sweep rate, MW power, and wait time in the pump-probe measurement, the nuclear field is also sensitive to the set value of i. the bias voltage V_SD_, ii. the dot detuning ε, iii. the inter-dot tunneling strength (reflected by the spin-conserving tunneling matrix element t_N_ and the spin-flipping tunneling matrix element t_F_), and iv. the coupling strength of the left (right) QD to the source (drain) contact Γ_L_ (Γ_R_). Set values for quantities i. to iv. are determined by voltages applied to the electrodes. The parameter space is very large. The bidirectional pumping effect we report here is not restricted to a unique combination of voltages applied to the electrodes. We considered that the most insightful parameters to vary for this initial investigation of the bidirectional pumping effect are the B-field sweep rate, MW power, and wait time in the pump-probe measurement.

### Outline of model for the bidirectional dragging and nuclear polarization effect

We briefly outline our model for the bidirectional dragging and nuclear polarization effect which we develop further in Section II of the Supplementary Note. The finite spin-flip tunneling of holes due to the SOI has the following important consequence. In the regime where the detuning is sufficiently large, the excitation GS→ES1 is spin-like rather than charge-like^1,29^, and the GS is basically hole spin-down ($|\downarrow \rangle$), whereas the ES1 is primarily hole spin-up $\left(A|\uparrow \rangle +b|\downarrow \rangle \right)$ with $\left|A\right|\gg \left|b\right|$⊡ However, $b$ cannot be ignored, and plays a vital role in the explanation of the bidirectional nuclear polarization phenomenon (as well as the electrical tunability of g_eff_). The SOI, in concert with the hyperfine interaction, creates a Knight field resulting in a nuclear spin quantization axis that is different for a hole in the first-excited-state-manifold compared to a hole in the ground-state-manifold. The tilted nuclear spin quantization axis in the hole-first-excited-state-manifold leads to a finite probability for nuclear spin transitions that can be described by a symmetric spectral function. As a consequence, nuclear spins can be flipped equally up or down depending on from which side the bare EDSR condition is approached (either on sweeping the B-field at constant detuning, or sweeping the detuning at constant B-field). The nuclear polarization leads to an Overhauser shift whilst from the opposite perspective, the spin of the single hole acts as a small nano-magnet for the nuclei leading to a Knight shift so providing a feedback mechanism. We strongly stress that in the absence of the SOI, we would not observe nuclear spin pumping since the MWs themselves do not interact with the nuclei, i.e., the bare EDSR process conserves nuclear spin.

In our model, the spectral function describing the amplitude of possible transitions between the hole-ground-state-manifold and the hole-first-excited-state-manifold depends on the detuning ε reflecting the SOI. However, the spectral function does not depend on the MW power which controls the amplitude in the oscillation of the effective (GS-ES1) Zeeman energy gap for holes (Δε_Zeeman_) in the MW excitation process through change in the detuning Δε. Figure 2a reveals three regimes. In regime I, the MW power is sufficiently low that Δε_Zeeman_ is much smaller than the half-width of the spectral function (Δw) so only a single spin-flip transition is visited and hence the pumped nuclear field is small. In regime II, Δε_Zeeman_ becomes larger (but still with Δε_Zeeman_ < Δw) so multiple spin-flip transitions (all flipping the nuclear spin in the same direction) are involved and the nuclear field is more strongly pumped. In this regime, in the simplest terms, the increased MW power increases the rate at which the nuclear spin-flip transition happens in the MW absorption. This leads to an increased probability of pumping, and therefore a larger nuclear field, i.e., the stronger one pumps, the stronger the pumped nuclear field. In regime III, at high MW power, Δε_Zeeman_ becomes larger than Δw and the nuclear field is inefficiently pumped since transitions flipping the nuclear spin in opposing directions are visited and so the maximum value of B_N_ is diminished. Note that although Δε_Zeeman_ is related to Δε, the relationship is not linear and this non-linearity is the key ingredient as to why we can distinguish regimes I, II, and III.

### Characteristics of EDSR dragging

On repeatedly performing B-sweeps for an identical sweep protocol we find the EDSR signal is quite similar from sweep-to-sweep with some fluctuation ( ~1–2 mT) in position of the rising and trailing edges (Supplementary Fig. 6). The trends we observe in B_N_ with MW power and B-field rate have qualitative similarity with those reported in ref. ^30^ for unidirectional dragging in a DQD confining electrons, although the EDSR signal in our case is notably less variable from one sweep to another.

For the measurement shown in Fig. 2c, at the end of each sweep, the MWs remain on for a period of 60 s, and then the MWs are turned off for 10 s before the commencement of the following sweep. During this idle stage, the nuclear field starts to decay slowly towards zero. A small decay of the nuclear field crucially enables the bidirectionality seen here. On the up-sweep, for example, the MW-enabled transitions between the hole-ground-state-manifold and the hole-first-excited-state-manifold driving a negative change in B_N_ are on the high-energy side of the spectral function. A small decay of the nuclear field shifts the center of the spectral function so that the MW-enabled transitions between the hole-ground-state-manifold and the hole-first-excited-state-manifold required to drive a positive change in B_N_ on the down-sweep are now on the low-energy side of the spectral function. The decay enables the locking condition to be caught on the appropriate side of the spectral function to support bidirectional dragging.

### Nuclear polarization and nuclear spin relaxation

Our estimation of the nuclear field for 100% nuclear polarization is derived from equations and tabulated information in refs. ^4,41^. Explicitly, the total nuclear field ${B}_{N}=\,\sum _{i}{B}_{N}(i),$ where ${B}_{N}(i)$ is the nuclear field for each of the isotopes (indexed i) in the GaAs material hosting the quantum dots. Furthermore, for each of the isotope species, ${B}_{N}\left(i\right)=\,{\nu }_{i}{A}_{\parallel }^{i}{I}/g{\mu }_{B}$, where ${\nu }_{i}$, ${A}_{\parallel }^{i}$, $I$, $g$ and ${\mu }_{B}$ are respectively the isotope abundance, appropriate hyperfine coupling constant, nuclear spin, appropriate hole g-factor and the Bohr magneton. Our estimated value for the nuclear field of ~115 mT for 100% nuclear polarization for heavy-holes is determined with well established numbers of I = 3/2 for each of the ^69^Ga, ^71^Ga, ^75^As isotopes in GaAs with abundances 0.3, 0.2 and 0.5 respectively. For $g$, we use the bulk-effective (out-of-plane) g-factor g*=1.4 for heavy-holes (see also Supplementary Fig. 1). The final ingredients are the values for ${A}_{\parallel }^{i}$ for holes. These values are less well established. We take values from Table I in ref. ^41^ derived from first principles calculations. Numbers given in the text for attained pumped nuclear (Overhauser) fields should be taken as estimated order of magnitude values.

We consider that spin diffusion will most likely be the dominant mechanism for nuclear spin relaxation limiting T_1_. In general, the Zeeman energy of the nuclei is about 2000 times smaller than that of the carriers (here holes). At any finite magnetic field of the order of 1 T it is not possible energetically for holes to “flip-flop” with the nuclei because the energy scales do not match. We stress we can achieve pumping of nuclear spins only because we are delivering the “missing” energy with microwave photons. The reverse channel (by emission rather than resonant absorption of microwaves) is unlikely to be dominant because there is no mechanism in place to account for the energy imbalance and thereby stimulate it.

### Microwave burst experiment and periodic oscillation of EDSR signal in time at Larmor frequency of ^75^As

The protocol implemented in the experiment is shown in the schematic in Fig. 4a. At constant B-field, *deep* in the energy blockade regime where one spin-down hole is trapped in the right QD, gate voltages V_L_ and V_R_ are changed *slowly* so that the resultant change in gate voltage is parallel to the edge of the transport triangle, and the detuning ε is swept from *high to low positive* detuning (solid blue arrow in right cartoon). Because of the SOI and consequent voltage-dependent g-factor such a sweep in detuning has similarity to adjusting the B-field at constant detuning (see plot of energy gap E_GS-ES1_ in Fig. 1d). Concurrently, the DQD is subjected to a continuous sequence of steps. One cycle with *fixed* period T = 1 μs of Initialization-Manipulate-Wait-Read-Idle steps is depicted in the left cartoon. In each cycle, there is a MW burst of duration t_burst_ (Manipulate) applied to coherently manipulate the trapped hole in the right QD *if the EDSR condition is attained* in which case the hole may be excited from the GS (spin-down) to ES1 (a mixture of spin-up and spin-down but predominantly spin-up). If the EDSR condition it met, the spin of the trapped hole starts to rotate from spin-down towards spin-up leading to population transfer and a mixed spin state. After the MW burst, during a period of time τ (Wait), an excited hole that is still trapped on the right QD can interact with the nuclear ensemble. During this step, the SOI acting with the hyperfine interaction causes the nuclear spin quantization axes of the hole-first-excited-state manifold to tilt away from the nuclear spin quantization axis of the hole-ground-state manifold, i.e., a Knight field that is perpendicular the external B-field is present, and the nuclear spins evolve which can lead to nuclear polarization. Subsequently the hole spin state is read out (Read). This step is achieved by momentarily pulsing the gate voltages for a period of time t_readout_ to increase the energy of the single-hole levels in the right QD such that ES1, but not GS, is raised above the Fermi energy in the drain contact (step depicted by blue dashed line cutting the red EDSR line feature in the right cartoon). A hole (predominately spin-up) in ES1 can tunnel to the drain contact (D) whereas a hole (spin-down) in GS1 stays in the right QD. For the former case, there is a contribution to the current signal. The signal also reflects any built-up nuclear polarization. Successful readout empties the right QD, the Knight field is switched off, and the evolution of the nuclear ensemble is quenched. During the subsequent Idle and Initialization (Init.) steps a down-spin hole is reloaded into the right QD before the next MW burst. The initialization time is fixed, but the idle time is varied to ensure one Initialization-Manipulate-Wait-Read-Idle cycle is of constant duration T = 1 μs. The DC current I is measured with an averaging time of ~0.1 s, i.e., the number of cycles per data point is ~10^5^. See Section II of the Supplementary Note for further details of role of each step in the sequence and how the steps impact the proposed mechanism for the periodic oscillations in the EDSR signal.

In summary, after MW excitation (the Manipulate step of pulse sequence), dependent on whether a hole in the DQD is a molecular excited state during the Wait step or is in the ground state localized on the right dot during the subsequent Read step, the nuclear spin quantization axis is periodically tilted away from or aligned parallel to the external B-field. This “rocking” motion of the nuclear spin quantization axis acts as an effective oscillating Knight field. In the experiment in Fig. 4, the oscillation is forced by the repetitive MW burst applied to gate electrode R during the Manipulate step and the repetitive elevation of the excited state (but not the ground state) above the Fermi energy in the drain contact during the Read step by pulsing voltages applied to the gate electrodes. The Manipulate step promotes a trapped hole from a localized ground state to a delocalized excited state, and the Read step removes a hole in the delocalized excited state from the DQD system and replaces that hole with a fresh hole from the source contact and ensures it is trapped on the right dot.

The difference in detuning between the maximum and minimum dragging position in Fig. 4b is ~180 μeV. From the corresponding change in the energy gap E_GS-ES1_ at ~1 T, the effective change in the hole g-factor is estimated be ~0.002 or equivalently B_N_ is ~1.5 mT.

Figure 4d shows a compilation of the EDSR signal position in detuning (arb. units) as a function of τ extracted from five data sets. The data set for B = 0.988 T is presented in Fig. 4b. The data sets for B = 0.909 and 1.068 T are presented in Supplementary Fig. 8. We note that because of non-uniform transmission of the MWs in the coaxial lines with frequency, the MW power is not the same for all five traces given in Fig. 4d. For the f = 19.46 GHz (B = 0.988 T) case, transmission is particularly high hence a much-reduced MW power has been used. Furthermore, although the nominal MW burst time t_burst_ is 100 ns, because of practical pulse timing and pulse delay effects, the influence of the MW burst spills into the start of the wait part of the pulse. Hence in Fig. 4d we have excluded the data between τ = 0 ns and τ = 70 ns (see Supplementary Fig. 8 where distortions are more evident at small τ). Finally, the phase of the oscillations in Fig. 4d is arbitrary in this experiment. We speculate that the nuclear spins acquire some arbitrary phase during the MW burst which may be power-dependent.

We do not observe oscillations commensurate with the Larmor precession of ^69^Ga and ^71^Ga nuclei in our data. This we attribute to the lower relative abundances, and the near factor of ten smaller valence-band hyperfine parameters (${A}_{\parallel }$) as compared to ^75^As tabulated in ref. ^41^.

## Supplementary information

Supplimentary Information_clean copy_23052025

## Data Availability

All relevant data and figures supporting the main conclusions of the document are available on request. Please refer to Sergei Studenikin at sergei.studenikin@nrc-cnrc.gc.ca.

## Code availability

All relevant code supporting the document is available upon request. Please refer to Sergei Studenikin at sergei.studenikin@nrc-cnrc.gc.ca.
