# Supplementary material for: Bidirectional nuclear polarization through electric dipole spin resonance enabled by spin-orbit interaction in a single hole planar quantum dot device
Source: npj Quantum Inf. 2025 Aug 7;11(1):133. doi: 10.1038/s41534-025-01075-0 (PMC12331515; doi:10.1038/s41534-025-01075-0)
Supplement: Supplementary file 1 — Supplimentary Information_clean copy_23052025 [file 41534_2025_1075_MOESM1_ESM.pdf]

Supplementary Information to:
"Bidirectional nuclear polarization through electric dipole spin resonance enabled by spin-orbit interaction in a single hole planar quantum dot device"

Authors: Sergei Studenikin[1*], Jordan Ducatel[1], Olivia Ellis[1,2], Marek Korkusinski[1,2], Alex Bogan[1], Piotr Zawadzki[1], D. Guy Austing[1*] & Andrew Sachrajda[1]

[1]Emerging Technologies Division, National Research Council of Canada, Ottawa, ON K1A0R6, Canada.
[2]Department of Physics, University of Ottawa, Ottawa, ON K1N6N5, Canada.

[*]e-mail: sergei.studenikin@nrc-cnrc.gc.ca; guy.austing@nrc-cnrc.gc.ca

Date: 23 May 2025

CONTENTS:

Supplementary Figures 1-9

Supplementary Note I: Transport triangle and EDSR line feature

Supplementary Note II: Simple model for bidirectional nuclear polarization
A. Introduction
B. Impact of strong SOI leading to different nuclear spin quantization axes in the hole-ground-state manifold and in the hole-first-excited-state manifold
C. Bidirectional dragging and nuclear polarization
D. Microwave burst experiment and periodic oscillation of EDSR signal in time

References

Supplementary Figures 1 to 9

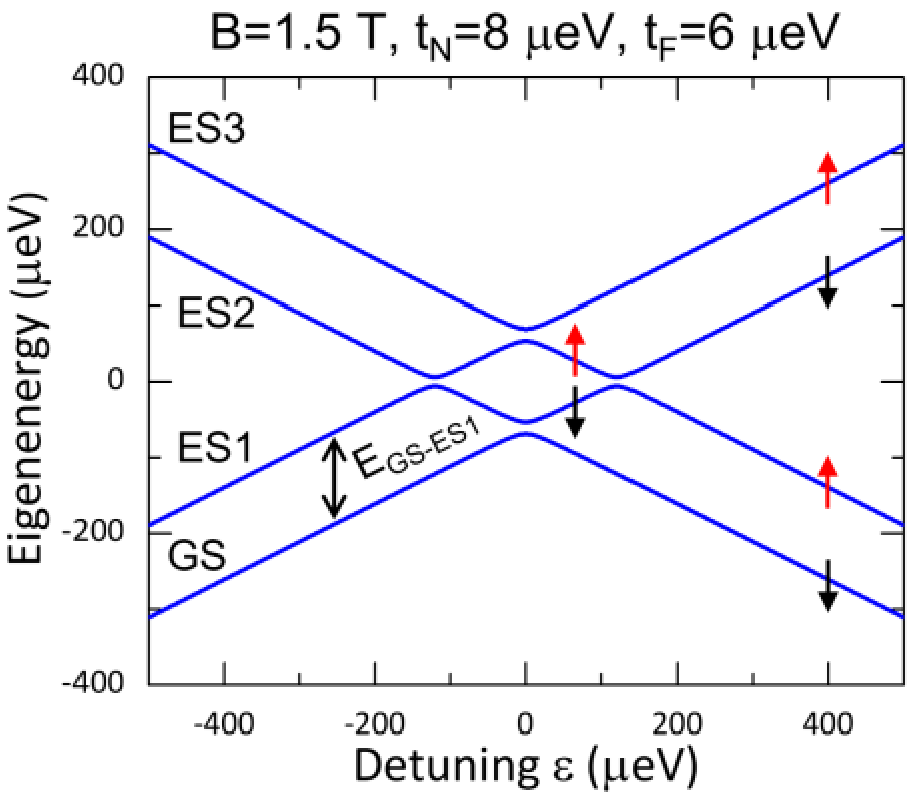

Supplementary Fig. 1 Calculated eigenenergies of the four lowest single-hole levels (ground state, GS, three excited states, ES1, ES2, and ES3) as a function of detuning ε at B=1.5 T. The hole spin is indicated by black and red arrows for positive detuning. Parameters: spin-conserving tunneling matrix element t_N=8 μeV, spin-flipping tunneling matrix element t_F=6 μeV, bulk-effective g-factor g*=1.4. For the parameters employed here relevant to Fig. 1b in the main text, note that at positive detuning ES1 and ES2 anti-cross (due to the presence of finite SOI) near ε~120 μeV where they are separated in energy by $2t_F$. Furthermore, to the left (right) of this anti-crossing the transition across the between GS and ES1 has charge-like (spin-like) character. In our experiments of the bidirectional dragging of the EDSR condition we operate in the regime where the detuning is sufficiently large at positive detuning that the GS is essentially hole located on the right QD spin-down $|R\downarrow\rangle$, whereas the ES1 is *mostly* hole located on the right QD spin-up $(A|R\uparrow\rangle + b|L\downarrow\rangle)$ with $|A| \gg |b|$. Nonetheless b cannot be neglected, and this component is essential for the explanation of the phenomenon.

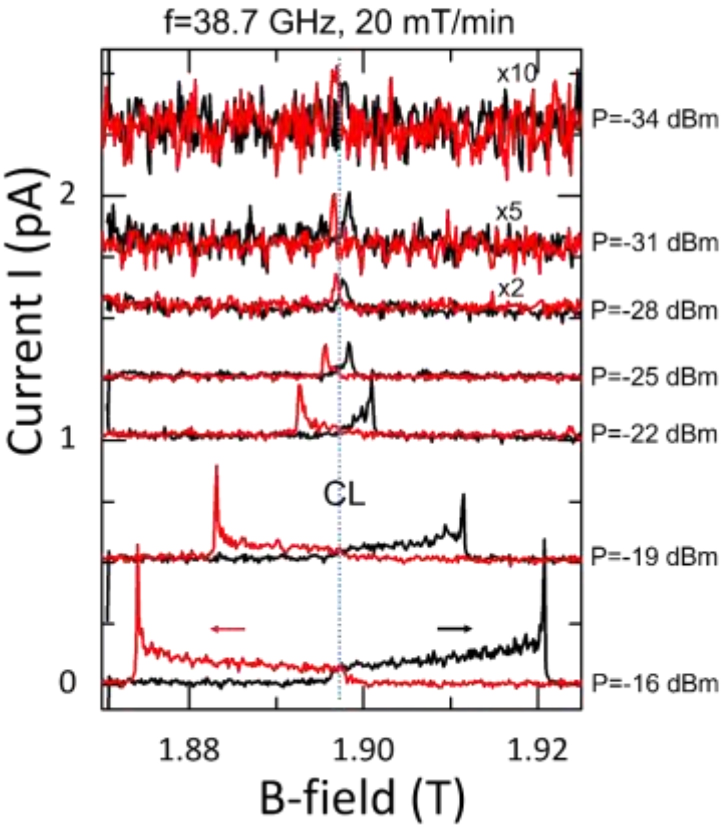

Supplementary Fig. 2 Full data set showing EDSR signal for various powers between -34 and -16 dBm. I is measured as a function of B with f=38.7 GHz. The B-field is swept up (black trace) and down (red trace) at rate 20 mT/min. The traces are vertically offset by different values relative to the P=-16 dBm traces for clarity. The current for the P=-34, -31, and -28 dBm traces respectively has been magnified by factors of 10, 5, and 2. The vertical dashed line CL marks the effective B-field position of the pure EDSR peak in the absence of dragging (the average position over all traces is B=1.897 T). The EDSR signal is just visible above the noise level in the P=-34 dBm traces and the nuclear field is ~0.5 mT, i.e., even at the lowest MW power for which we can observe EDSR small remnant hysteresis due to some nuclear pumping is still present. The EDSR linewidth (full width at half maximum) ~1 mT for the P=-34 dBm traces is the smallest we observed. The maximum nuclear field here for the P=-16 dBm traces is ~23 mT. Note also that for mid-range power, the rising edge of the EDSR signal starts up to ~5mT before the expected position of the pure EDSR peak which attribute to the width of the spectral function. For this data set, V_{SD}=0.7 mV and ε~490 μeV. The measurement protocol involves sweeping the B-field up and down between 1.87 and 1.93 T. At the end of the up- (down-) sweep, the MWs are turned off for 600 s before the commencement of the following down- (up-) sweep with the MWs turned on at the same (next) power level.

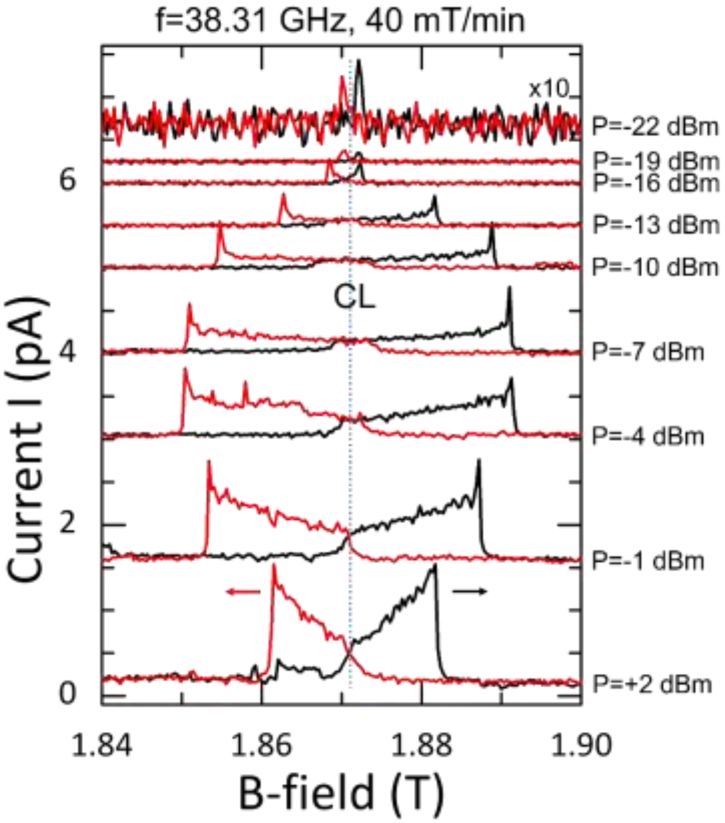

Supplementary Fig. 3 Full data set showing EDSR signal for various powers between -22 and +2 dBm. I is measured as a function of B with f=38.31 GHz. The B-field is swept up (black trace) and down (red trace) at rate 40 mT/min. The traces are vertically offset by different values relative to the P=+2 dBm traces for clarity. The current for the P=-22 dBm traces has been magnified by a factor of 10. The vertical dashed line CL marks the effective B-field position of the pure EDSR peak in the absence of dragging (the average position over all traces is B=1.871 T). The nuclear field for the P=-22 dBm traces is ~1 mT. The maximum nuclear field here for the P=-4 dBm traces is ~20 mT. At higher MW power the maximum nuclear field achieved is diminished. For this data set, V_{SD}=0.5 mV and ε~470 μeV. The measurement protocol involves sweeping the B-field up and down between 1.82 and 1.94 T. At the end of the up- (down-) sweep, the MWs remain on and the following down- (up-) sweep commences immediately with the MWs at the same (next) power level. Note that in comparison to the data in Supplementary Fig. 2, the data set here extends to higher MW power.

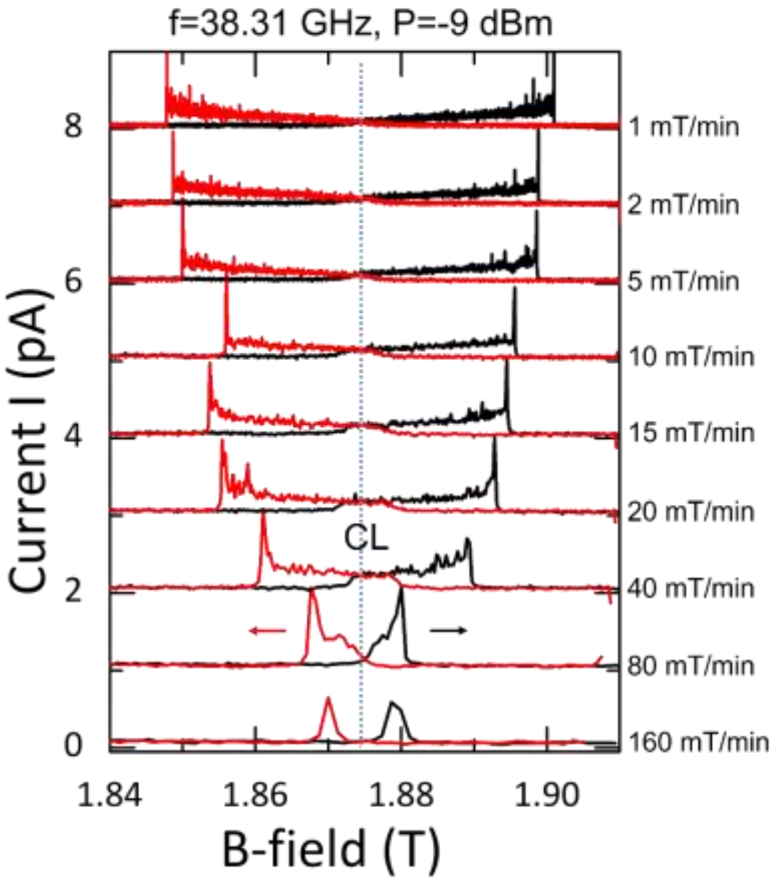

Supplementary Fig. 4 Full data set showing EDSR signal for various B-field sweep rates between 1 and 160 mT/min. I is measured as a function of B with f=38.31 GHz. The B-field is swept up (black trace) and down (red trace) at fixed MW power P=-9 dBm [in regime II as identified in Fig. 2a in the main text]. The traces are vertically offset relative to the 160 mT/min traces for clarity. The vertical dashed line CL marks the effective B-field position of the pure EDSR peak in the absence of dragging (the average position over all traces is B=1.874 T). The maximum nuclear field here for the 1 mT/min traces is ~26 mT. The maximum nuclear field achieved diminishes with B-field sweep rate. The nuclear field attained for the 120 mT/min traces is ~4 mT. For this data set, V_{SD}=0.5 mV and ε~210 μeV. The measurement protocol involves sweeping the B-field up and down between 1.83 and 1.91 T. At the end of the up- (down-) sweep, the MWs remain on and the following down- (up-) sweep commences immediately with the MWs at the same (next) power level. The EDSR line shape changes from triangular with a sharp peak and drop-off on termination at low sweep rate to peak-like at high sweep rate.

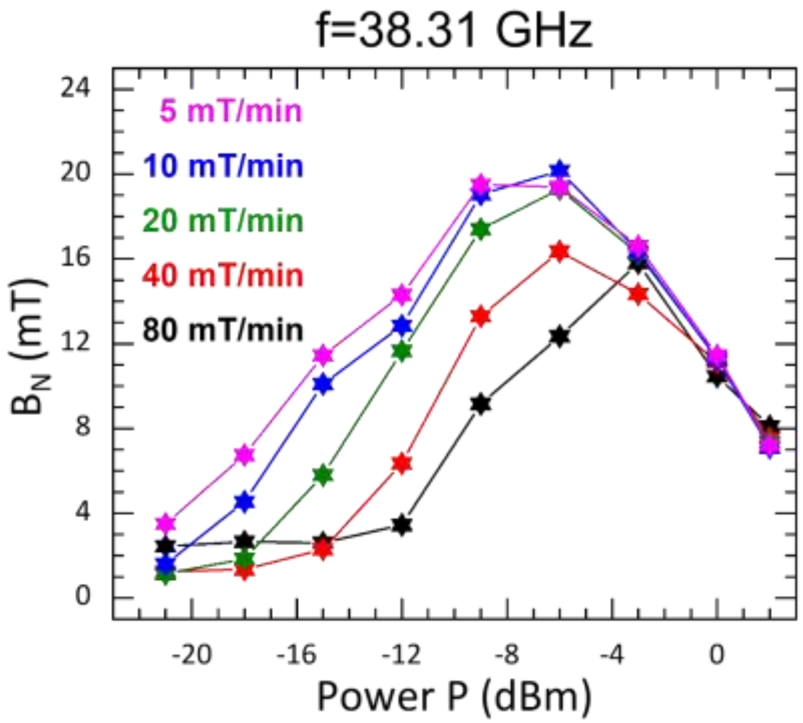

Supplementary Fig. 5 Compilation of maximum B_N from an extensive set of measurements for MW powers between -21 and +2 dBm for different B-field sweep rates between 5 and 80 mT/min. For set MW power and B-field sweep rate, B_N is estimated from both the up-sweep and down-sweep traces. The MW frequency is f=38.31 GHz. For the data set, V_{SD}=0.5 mV and ε~270 μeV. The measurement protocol involves sweeping the B-field up and down between 1.85 and 1.90 T. At the end of the up- (down-) sweep, the MWs remain on and the following down- (up-) sweep commences immediately with the MWs at the same (next) power level. The DQD parameters are slightly different from those for the data shown in the main text. Here, at 1.9 T, t_N is ~12 μeV, and t_F is ~7 μeV, i.e., the inter-dot couplings are slightly larger. Γ_L and Γ_R are also slightly different but still set such that $\Gamma_L >> \Gamma_R$. We observe the following trends: (i) up to P~4 dBm (the boundary between regimes II and III), at a given MW power, the maximum B_N generally increases as the B-field sweep rate is reduced; (ii) the boundary between regimes I and II moves to lower power also as the B-field sweep rate is reduced; and (iii) beyond P~4 dBm, the maximum B_N are very similar for the different B-field sweep rates.

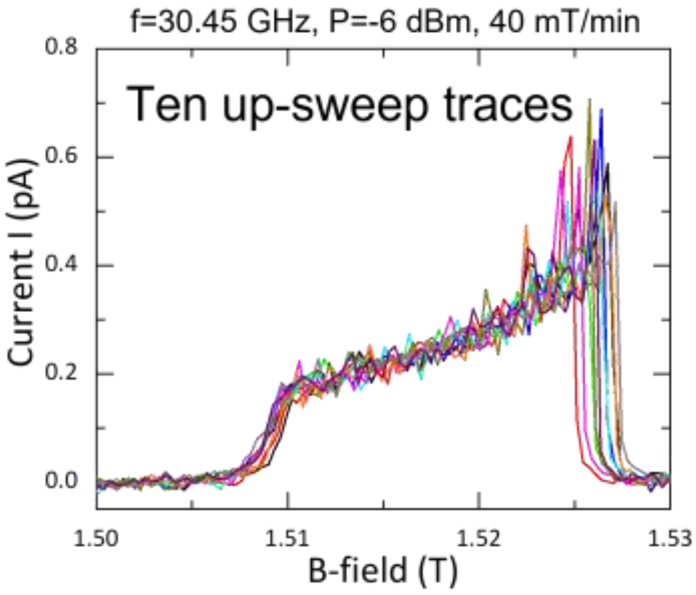

Supplementary Fig. 6 Variance in EDSR signal for repeated sweeps. Here we show ten up sweep traces. In each case, the B-field is swept from 1.495 to 1.53 T at rate 40 mT/min, and the MWs are on with f=30.45 GHz and P=-6 dBm. After each up-sweep the B-field is reset to 1.495 T under identical conditions, and after a 5 min wait with the MWs off, the following up-sweep commences. For the data set, V_{SD}=0.5 mV and ε~500 μeV. The EDSR signals are quite similar. The rising (trailing) edges of the ten traces are subject to some fluctuation in B-field position with spread of ~0.9 mT (~2.3 mT).

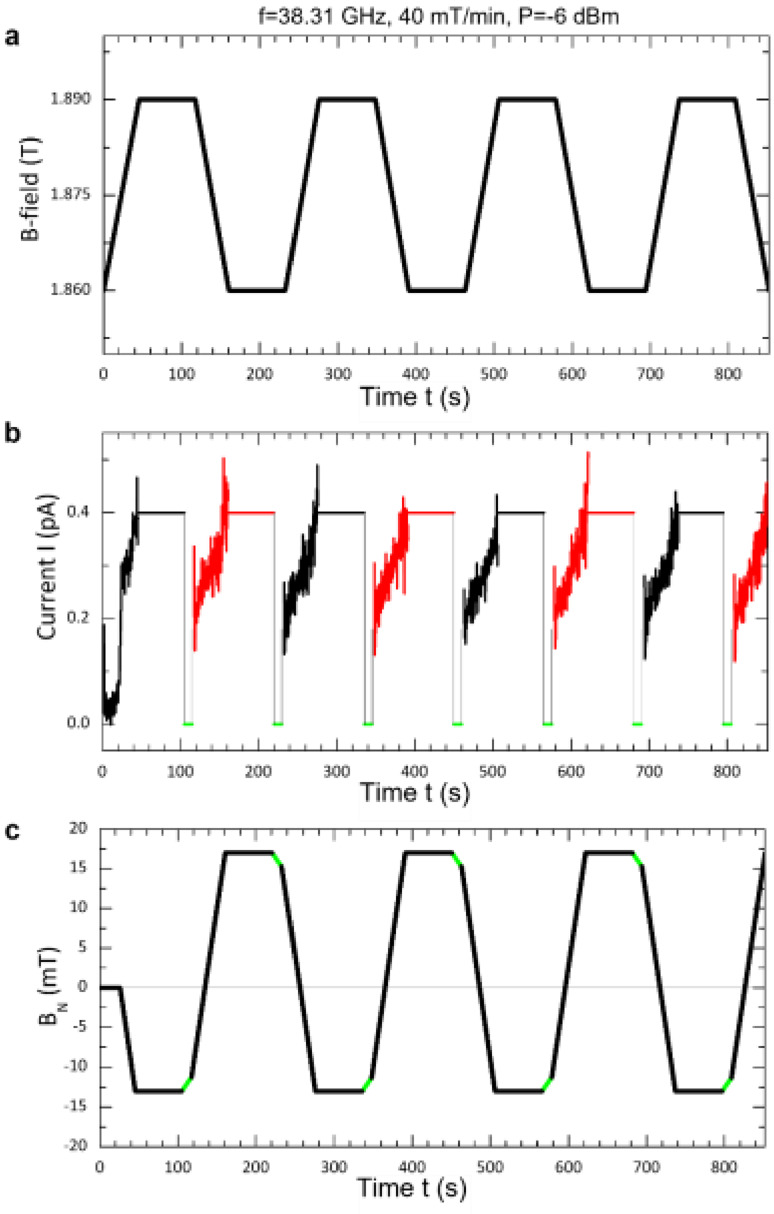

Supplementary Fig. 7 Locking experiment. In the main text, the first example of locking is based on an experiment whereby the B-field is swept up and down repeatedly, and other than at the start of the initial up-sweep (and when the MWs are momentarily turned off) resonance is maintained dynamically throughout. Here we replot the same data set as a function of time. **a** shows the applied B-field as a function of time t. **b** shows I as a function of t. Up-sweep (down-sweep) portions are colored black (red). At the end of each sweep the MWs remain on for a period of 60 s. I is not recorded in this interval but remains high (on-resonance). Subsequently, the MWs are turned off for a period of 10 s. I is not recorded in this short interval since there is no EDSR signal. **c** shows the reconstructed nuclear field B_N attained during dragging

as a function of t. Other than the initial up-sweep, B_N changes from +15 to -10 mT on up-sweeps, and from -10 to +15 mT on down-sweeps). Note that the inferred position of the pure EDSR peak in the absence of nuclear polarization is ~2-3 mT to the right of the onset of finite current at ~1.875 T in Fig. 2c in the main text. Consequently, the 1.86 T to 1.89 T window of operation is slightly off-center and this explains why in the experiment the absolute values of B_N at 1.86 T and 1.89 T differ. After the EDSR condition is attained on the first up sweep, *other than when the MWS are turned off*, B_N changes dynamically in such a way as to compensate exactly the change in the B-field. When the MWs are turned off B_N decays starts to decay towards zero.

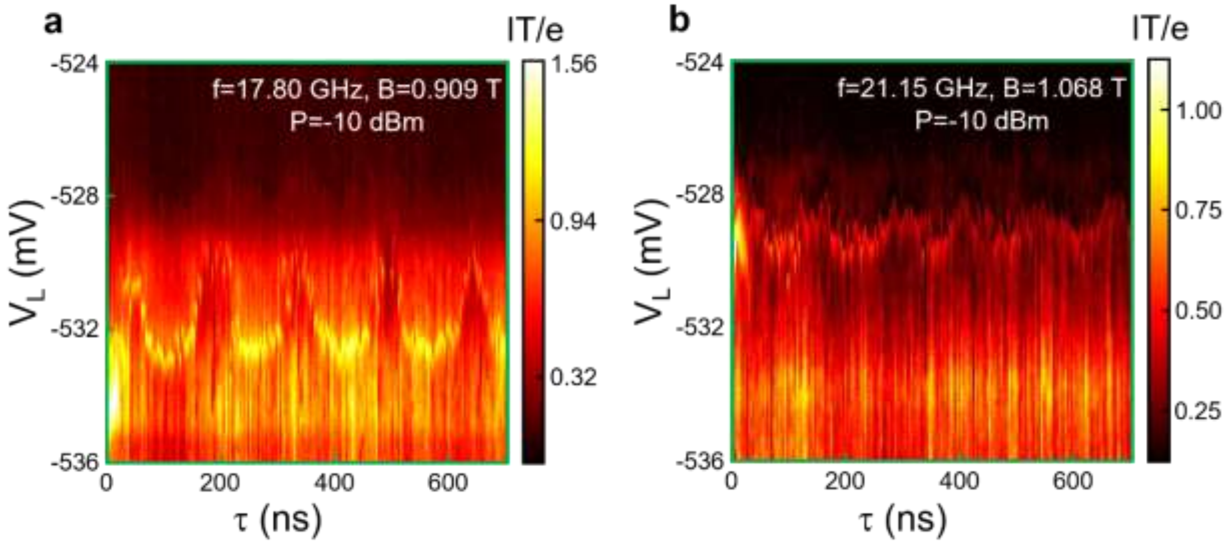

Supplementary Fig. 8 Other microwave burst experiments. In addition to the colour plot of current measured as a function of swept V_L (corresponding to detuning) and stepped τ at B=0.988 T with the MW frequency and power respectively set to f=19.46 GHz and P=-28 dBm given in Fig. 4 in the main text, we show here two other data sets. In **a** B=0.909 T, f=17.80 GHz, and P=-10 dBm, and in **b** B=1.068 T, f=21.25 GHz, and P=-10 dBm. Note that the current is also given in terms of IT/e (without subtracting the non-resonant background current), and for these experiments, V_{SD}=0.5 mV, t_{burst}=$t_{readout}$=100 ns, and the initialization period is 100 ns. Note that the oscillations in the EDSR signal position in both plots are distorted at small τ. We attribute this to practical pulse timing and pulse delay effects that cause the influence of the MW burst to spill into the start of the wait part of the pulse.

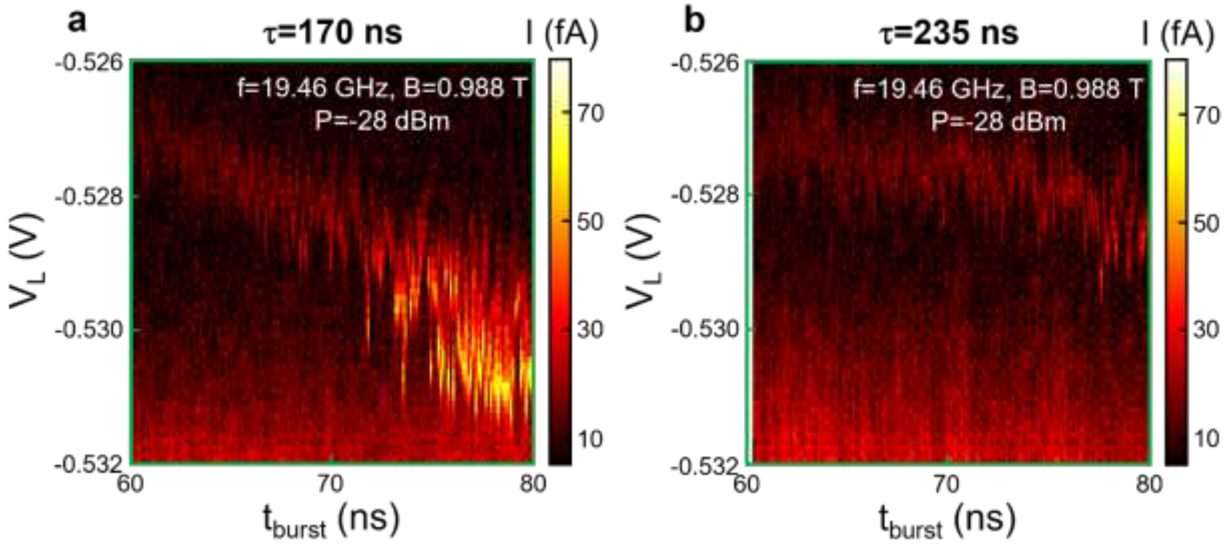

Supplementary Fig. 9 t_{burst} dependence with wait time τ set to maximal and minimal dragging conditions. Figure 4b of the main text shows periodic oscillations in the EDSR signal position in detuning as a function of stepped τ *for fixed* t_{burst}*=100 ns*. Here, also for B=0.988 T and with the MW frequency and power respectively set to f=19.46 GHz and P=-28 dBm, we show for the same pulse sequence the EDSR signal evolution as a function of stepped t_{burst} with τ=170 ns (**a**) [close to a maximal dragging condition: see Fig. 4c], and with τ=235 ns (**b**) [close to a minimal dragging condition: see Fig. 4c]. As expected, in **a** the EDSR signal clearly grows in amplitude and shifts position significantly (to less positive detuning), whereas in **b**, the EDSR signal remains weak and hardly shifts in position. For these experiments, V_{SD}=0.5 mV, T=1 μs, $t_{readout}$=100 ns, and the initialization period is 100 ns. t_{burst} here is the nominal duration of the MW burst. However, due to instrumentation there is not an exact correspondence between the set value and the actual value of t_{burst} output from the microwave signal generator. After calibration a set value of 60 ns (80 ns) is found to give an actual value of ~60 ns (100 ns). If set to a maximum dragging condition one might anticipate oscillations in the EDSR signal position with t_{burst} reflecting Rabi oscillations of the hole spin, however, the experimental conditions pertinent to these particular measurements are not optimal for the observation of such oscillations (we are far from even a π-pulse).

Supplementary Note I: Transport triangle and EDSR line feature

Figure 1 (b) of the main text shows an example of a transport triangle also referred to in the literature as a bias triangle [1-3]. Here we provide extra commentary on the transport triangle and the EDSR line feature. In Supplementary Fig. 10 we show the data in Fig. 1 (b) of the main text replotted with extra labelling of features for guidance. As well as plotting data as the derivative of the current with respect to gate voltage V_L, we additionally show the current (in both low and high contrast plots), along with a section through the EDSR line feature.

In Supplementary Fig. 10 (a) we have marked the two long sides of the transport triangle under focus with dashed cyan lines. Part of the adjoining transport triangle is visible in the bottom left corner of the plot. The solid cyan lines identify the underlying hexagonal or so-called "honeycomb" lattice [1, 2], and the green circles mark the triple points ("anchor points" for the transport triangles). For very small bias voltage (V_{SD}), the transport triangles would shrink to the triple points and one would recover a familiar charge stability diagram [1, 2]. The relevant cycle here for hole transport through the DQD leading to a current at finite bias voltage is $(N_L,N_R)=(0,0)\rightarrow(1,0)\rightarrow(0,1)\rightarrow(0,0)$ where $N_L(N_R)$ is the hole occupation on the left (right) dot. In the absence of MWs, outside of the transport triangle of focus, no current flows and the DQD is Coulomb blockaded. We have labeled the appropriate Coulomb blockaded regions by the trapped hole configurations (0, 0), (1,0), and (0,1). See Refs. [2, 7] for a discussion on how we confirm here that N_L and N_R are absolute occupation numbers rather than effective occupation numbers. In the presence of MWs, for the measurements reported in the main text, at positive dot detuning [see Fig. 1 (c) in the main text], an EDSR line feature (marked by the white arrow) appears just outside the transport triangle when MWs enable the GS$\rightarrow$ES1 transition (Supplementary Fig. 1). The EDSR line feature starts at the edge of the transport triangle, extends outwards into the otherwise Coulomb blockaded region, and then terminates at a point on the marked dashed yellow line. The separation between the edge of the transport triangle (dashed cyan line) and the parallel running dashed yellow line reflects the Zeeman energy gap for holes controlled by the external B-field [3, 4]. We stress that for the measurement here, the goal is to demonstrate the presence of the EDSR line features but not strongly pump the nuclear, i.e., the dragging of the EDSR signal is not apparent in the data.

In Supplementary Fig. 10 (b), the current I rather than the derivative dI/dV_L is plotted as a function of the voltages V_L and V_R on the gates L and R respectively. The transport triangle is clear, and we have copied the solid and dashed cyan lines, the green circles, and trapped hole configuration numbers from panel (a) to facilitate easy comparison. The current scale is set to cover the range from 0 to 6 pA so this a low contrast plot and the weaker EDSR line feature is not evident. In Supplementary Fig. 10 (c), the current scale is set to cover the range 0 to 0.8 pA. In this high contrast plot the transport triangle color is saturated

and the triangular shape of the transport triangle appears distorted because at the sub-pA level second-order cotunneling processes [1, 2] present also become observable. Nonetheless, visible in panel (c), marked by the white triangle, is the EDSR line feature of interest. Inspecting the line cut through the EDSR line feature marked by the dashed green line, we can see the EDSR signal plotted in panel (d). For further general details of the EDSR line feature (in the absence of the bidirectional nuclear polarization effect), see Refs. [3, 4]. For a general introduction to transport through DQDs, see Refs. [1, 2].

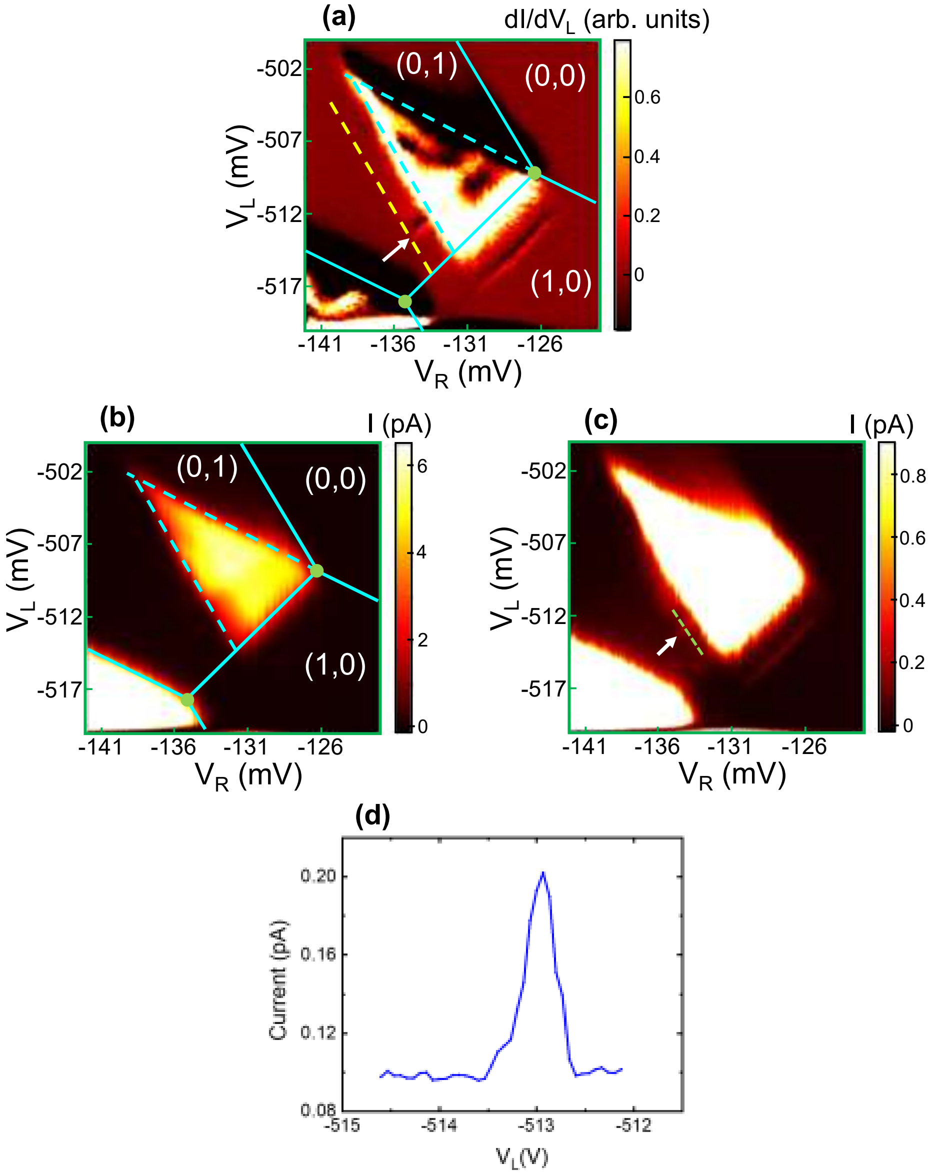

Supplementary Figure 10: Transport triangle and EDSR line feature. (a) Figure 1 (b) shown in the main text replotted with extra labelling of features for guidance. The same data set is also plotted showing the directly measured current in (b) low and (c) high contrast plots, along with a section (d) through the EDSR line feature. See accompanying text for discussion.

Supplementary Note II: Simple model for bidirectional nuclear polarization

A. Introduction

We sketch out key components of our simple model salient to the experiments described in the main text. A full theoretical description of our model will be described elsewhere [5]. For simplicity, in the following discussion, we shall assume the nuclei are all of one species; the nuclear spin of each nucleus is 1/2; the nuclei do not interact with each other; the total nuclear spin of the ensemble of nuclei is the vector sum of the individual nuclear spins; and that we only need to consider the projections of the total nuclear spin against the instantaneous total effective magnetic field direction- the quantum numbers M_Z and $\widetilde{M}_Z$ in the following.

As a preamble to the discussion in the following sections we would like to stress the following:

i. The hole states in our system are generally superpositions of heavy-hole (HH) and light-hole (LH) states, as in any zero-dimensional structure. However, we have previously demonstrated experimentally the content of the relevant hole-states to be predominantly heavy-hole [4]. In terms of theory, this stems from the heterostructure confinement in the growth direction, which discriminates between HHs and LHs due to their different mass, making the HHs lower in energy. From simulations, we expect that the LHs are actually not confined in the heterostructure. The strongest experimental proof of the dominance of the HHs is the anisotropy of the g-factor which falls to nearly zero for an in-plane magnetic field [4]. Hence forth in our model we shall assume we are dealing exclusively with HHs and for brevity we simply call them “holes”.

ii. The quantum confined states relevant to bidirectional nuclear polarization effect are those of a double quantum dot. Our model is developed taking into account just the two lowest energy states *in each dot*, one state with spin up and one state with spin down (in the following the left dot states labelled $|L\downarrow\rangle$ and $|L\uparrow\rangle$, and the right dot states labelled $|R\downarrow\rangle$ and $|R\uparrow\rangle$). These states are hybridized because of the finite height and width of the interdot barrier, and consequently quantum molecular states are formed. The mixing is due to both spin-conserving and spin-flipping tunneling [4]. The particular situation with the excited state that we are interested in is that one where the spin-down state from the left dot hybridizes with the spin-up state from the right dot.

iii. As well as the confined holes, there is also the nuclear subsystem. The full configurations of our hole-nuclear system are tensor products of the hole states and the states of the nuclei. The “manifolds” that we discuss are the Hilbert subspaces of that very large Hilbert space (of the hole and all the nuclei). The lower hole manifold (hence forth the “hole-ground-state manifold”) consists of the configurations involving a spin-down hole on the right dot in tensor product with all possible configurations of the nuclei, and the upper-hole manifold (hence forth “hole-first-excited-state manifold”) consists of the configurations

involving a *molecular* excited hole state (which does not have a definite spin since it is a superposition of hole spin up and hole spin down states) in tensor product with all the nuclear configurations. The two subspaces are very populous (they consist of many configurations) and that is why we call them "manifolds".

iv. A single quantum dot device is insufficient to observe the bidirectional nuclear polarization effect reported. A minimum of two coupled dots are necessary since *molecular* states are an essential ingredient. As will become clear in the following sections, the central property enabling nuclear spin pumping is the difference in alignment of spin quantization axes in the lower and upper manifolds of the states of the hole coupled to the nuclei. This is brought about by the difference in the direction of the hole spin axis, as this, via the hyperfine interaction, translates into the effective (Knight) magnetic field experienced by the nuclear spins. In a single quantum dot system, we can have the hole states with opposite spins, but these spin directions are really defined according to the same quantization axis. In the double quantum dot system we study, we can generate a condition whereby the hole state in the lower manifold is effectively a single-dot one (weakly hybridized), while the hole state in the upper manifold is a superposition of two single-dot hole states: spin down from the left dot and spin up from the right dot. That non-spin-conserving superposition is brought about by the spin-orbit interaction. Thus, we have engineered the difference in the hole spin quantization axes by bringing in the second quantum dot, without which the spin pumping would not have taken place.

B. Impact of strong spin-orbit interaction leading to different nuclear spin quantization axes in the hole-ground-state manifold and in the hole-first-excited-state manifold

In our model, basis states of the full system are composed of two-components: one component relating to the hole and the other component relating to the nuclear spin. For the pertinent experimental conditions, basis states in the hole-ground-state manifold are of the form $|R\downarrow\rangle|M_z\rangle$ since the hole is located on the right (R) quantum dot (QD) and spin-down. On the other hand, reflecting the influence of the spin-orbit interaction (SOI), basis states in the hole-first-excited-state manifold are of the form $(A|R\uparrow\rangle + b|L\downarrow\rangle)|M_Z\rangle$. Although the hole is still principally to be found on the R QD (adjacent to the drain) and spin-up, there is a small but non-negligible probability that the hole is found on the left (L) QD spin-down, i.e., $|A| \gg |b|$. The presence of a finite b plays a critical role in our interpretation of the experimental data. The coefficients A and b can be tuned by changing the dot detuning- see main text and Fig. 1 (d) and Supplementary Fig. 1. For both manifolds, $|M_z\rangle$ represents the z-component of the nuclear spin basis state.

In the absence of interactions between the hole and the nuclear spins, the (nuclear Zeeman) energy gap between consecutive levels within each of the two manifolds would be $g_N\mu_{B,N}B_z$ where g_N, $\mu_{B,N}$, and B_z respectively are the nuclear g-factor, nuclear magneton, and z-component of the magnetic field. In this

situation, since microwaves (MWs) do not "talk" to the nuclei, EDSR of a hole surrounded with (non-interacting) spectator nuclei must conserve nuclear spin, i.e., absorption is only possible between hole basis states in the two manifolds if they have the same $|M_z\rangle$, for example, $|R\downarrow\rangle|M_z=0\rangle$ → $(A|R\uparrow\rangle+b|L\downarrow\rangle)|M_Z=0\rangle$, and $|R\downarrow\rangle|M_z=+1\rangle$ → $(A|R\uparrow\rangle+b|L\downarrow\rangle)|M_Z=+1\rangle$, …. Note that the MW frequencies for all these transitions (of energy E_0) are identical because all the energy gaps between neighboring nuclear spin basis states in both manifolds are the same, and consequently the EDSR resonance condition is the same irrespective of the nuclear spin basis state.

Referring to Supplementary Fig. 11 (a), the picture changes significantly when interactions between the hole and nuclear spins are considered. Parallel to the external field there are Overhauser/Knight terms with strength $A_{||}^{(i)}$, and perpendicular to the external field there are spin exchange terms with strength $A_{\perp}^{(i)}$ [6]. Here *i* indexes the individual nuclei. For simplicity, we henceforth drop this index, i.e., we apply the "top-hat" approximation and assume the hole is equally coupled to all nuclear spins in the DQD.

Focusing first on the longitudinal Overhauser/Knight component, we assume $A_{||}<0$. The hole spin acts as an extra magnetic field for the nuclei. In the hole-ground-state manifold the energy gap between consecutive basis states becomes $g_N\mu_{B,N}B_z+|A_{||}|$, i.e., the effective nuclear Zeeman energy is *augmented* by the Knight term. In the hole-first-excited-state manifold the energy gap between consecutive basis states becomes $g_N\mu_{B,N}B_z-|A_{||}|(A-b)$, i.e., the effective nuclear Zeeman energy is *diminished* by the Knight term. Consequently, there is a profound impact on the EDSR resonance frequency. With $|M_z=0\rangle$, the EDSR frequency (of energy E_0) is the same as that without interactions because there is no net nuclear polarization. However, for $|M_z\neq 0\rangle$ the EDSR frequency is no longer the same *and* becomes dependent on the value of $|M_z\rangle$ itself, for example, for $|R\downarrow\rangle|M_z=+1\rangle$ → $(A|R\uparrow\rangle+b|L\downarrow\rangle)|M_Z=+1\rangle$ the EDSR resonance is at energy $E_0-2|A_{||}|$ (assuming $|A|\gg|b|$), $|R\downarrow\rangle|M_z=+2\rangle$ → $(A|R\uparrow\rangle+b|L\downarrow\rangle)|M_Z=+2\rangle$ the EDSR resonance is at energy $E_0-4|A_{||}|$, …, **namely the EDSR resonance frequency depends on nuclear polarization**.

Turning our attention to the spin exchange (transverse Overhauser/Knight) component, states within the low-energy manifold remain uncoupled because the hole spin is not a superposition: in this manifold, the hole spin is a good quantum number, and the nuclear spin is also a good quantum number (the M_Z quantum numbers are according to the external magnetic field). Consequently, the Knight field (B_{Knight}) is collinear with the external magnetic field (B_{ext}) taken to be parallel to the z-direction, i.e., the Knight field has no component perpendicular to B_{ext} ($B_{Knight,x}$=0) and the nuclei experience a total effective magnetic field which is directed just along the z-direction. The situation is different in the high-energy manifold because the hole-spin is not completely up and so spin exchange ($A_{\perp}$) is active, for example, $(A|R\uparrow\rangle+b|L\downarrow\rangle)|M_Z=0\rangle$ is coupled to both $(A|R\uparrow\rangle+b|L\downarrow\rangle)|M_Z=+1\rangle$ with strength A^*b and

$(A|R\uparrow\rangle + b|L\downarrow\rangle)|M_Z = -1\rangle$ with strength Ab^*. Equivalently, $(A|R\uparrow\rangle + b|L\downarrow\rangle)|M_Z \approx 0\rangle = \alpha(\ldots + A|R\uparrow\rangle + b|L\downarrow\rangle)|M_Z = -1\rangle + \beta(A|R\uparrow\rangle + b|L\downarrow\rangle)|M_Z = 0\rangle + \gamma(A|R\uparrow\rangle + b|L\downarrow\rangle)|M_Z = +1\rangle + \cdots)$ up to first-order with coefficients $\beta \sim 1 \gg \alpha, \gamma$, or factoring out the hole state, $(A|R\uparrow\rangle + b|L\downarrow\rangle)|M_Z \approx 0\rangle = (A|R\uparrow\rangle + b|L\downarrow\rangle)\,[\ldots + \alpha|M_Z = -1\rangle + \beta|M_Z = 0\rangle + \gamma|M_Z = +1\rangle + \cdots]$, i.e., the hole is untouched- it is the nuclei that adjust. Consequently, the Knight field (B_{Knight}) is no longer collinear with the external magnetic field, i.e., the Knight field has finite components parallel and perpendicular to B_{ext} and the nuclei experience a total effective magnetic field which is not directed just along the z-direction. $B_{Knight,x}$ can be shown to be proportional to $A_\perp$, A, and b. **Note that the nuclear spin states we have written as …, $|M_Z \approx -1\rangle$, $|M_Z \approx 0\rangle$, $|M_Z \approx +1\rangle$ … are essentially states in the tilted axis basis and henceforth we will write them in terms of new quantum numbers …, $|\widetilde{M}_Z = -1\rangle$, $|\widetilde{M}_Z = 0\rangle$, $|\widetilde{M}_Z = +1\rangle$, …, i.e., we will use notation $|M_Z\rangle$ ($|\widetilde{M}_Z\rangle$) for the basis states with the untilted (tilted) quantization axis in the low-energy (high-energy) manifold.**

Supplementary Fig. 11 (b) provides a physical interpretation. When the hole occupies the low-energy manifold the nuclear spin quantization axis is aligned with the external magnetic field B_{ext}. However, when the hole occupies the high-energy manifold, the SOI in combination with the hyperfine interaction induces a tilt of the nuclear spin quantization axis determined by the values $A_\perp$, A and b (larger SOI means larger b and consequently larger tilt). **In other words, because of the SOI the hole spin is tilted in the high-energy manifold and the tilt of the hole spin is then translated by means of the hyperfine interaction into the x-component of the Knight field which is felt by the nuclear spin**. The new nuclear spin quantization axis is now parallel to the total effective magnetic field which has both a z-component (B_{ext} - $B_{Knight,z}$) and a finite x-component ($B_{Knight,x}$).

For interpretation of the MW experiments, a further important consequence of the induced tilt of the nuclear spin quantization axis for the high-energy manifold is illustrated by referring to the spectral functions [7] in Supplementary Fig. 11 (a). Suppose we wish to track a specific state $|M_Z = \Lambda\rangle$. In the low-energy manifold, only one basis state has $|M_Z = \Lambda\rangle$: the spectral function will have non-zero amplitude for $|M_Z = \Lambda\rangle$ and zero amplitude for $|M_Z \neq \Lambda\rangle$. Now we pose the question: Which basis states in the high-energy manifold with tilted nuclear spin quantization axis can be accessed by MW transitions from the low-energy manifold state $|M_Z = \Lambda\rangle$? **Crucially in the EDSR process the nuclear spin polarization is conserved**. In the high-energy manifold, clearly the basis state $|\widetilde{M}_Z = \Lambda\rangle$ has the largest $|M_Z = \Lambda\rangle$ component and so the transition to $|\widetilde{M}_Z = \Lambda\rangle$ will be strongest. However, basis states $|\widetilde{M}_Z = \Lambda \pm 1\rangle$ also have a finite component of $|M_Z = \Lambda\rangle$ albeit of reduced weight and so the transitions to $|\widetilde{M}_Z = \Lambda \pm 1\rangle$ will be allowed but are weaker, and likewise transitions to basis states $|\widetilde{M}_Z = \Lambda \pm 2\rangle$ with even less weight to the component $|M_Z = \Lambda\rangle$ will be weaker still, and so on: the spectral function will be peaked for $|\widetilde{M}_Z = \Lambda\rangle$

but have a finite width. The spectral function is expected to be symmetric unless the nuclear polarization becomes significant (close to full polarization). In the illustrative example sketched in Supplementary Fig. 11 (a) the $|M_Z = 0\rangle$ state is tracked. MWs tuned to the set of $|R\downarrow\rangle|M_z = 0\rangle$ → $(A|R\uparrow\rangle + b|L\downarrow\rangle)\big|\widetilde{M}_Z = \cdots, -2, -1, 0, +1, +2, +3, \ldots\big\rangle$ transition can connect with the $(A|R\uparrow\rangle + b|L\downarrow\rangle)|M_Z = 0\rangle$ component with weight reflected by the Lorentzian-like spectral function depicted.

C. Bidirectional dragging and nuclear polarization

We now give a basic explanation of how dragging and nuclear spin pumping proceed. Supplementary Fig. 12 illustrates an oversimplified scenario that nonetheless shows the microscopic factors influencing the EDSR line shape. We focus on the case where the B-field is swept down from high to low field through the EDSR resonance (bold red trace in the current vs. B plot at the top of the figure). For points marked I-IX on the current vs. B trace, we discuss below the elemental processes involved, and in particular the roles of the spectral function and nuclear spin relaxation in determining the overall EDSR line shape. In the simple example we shall give, we will assume transitions $|M_Z = \Lambda\rangle$ → $\big|\widetilde{M}_Z = \Lambda \pm 2\big\rangle$ are the weakest to have an impact. The "tightness" of the allowed transitions, or equivalently the narrowness of the spectral function, reflects the condition $|A_\perp| \ll |A_{||}|$, i.e., the tilt of the nuclear spin quantization axis in the high-energy manifold is small.

We now outline specific situations at points I-IX depicted in Supplementary Fig. 12. (I) No current flows above the MW resonance (high B-field side) where "allowed" transitions (with non-negligible amplitude) do not occur because the effective (GS-ES1) Zeeman energy gap is too large. (II, III) On lowering the B-field, the effective Zeeman energy gap is reduced. The $|M_z = 0\rangle$ → $\big|\widetilde{M}_Z = -2\big\rangle$ transition is now allowed. Near the rising edge of the EDSR signal, there is a relatively large change in M_Z (leading to build up of nuclear polarization), here by a step of 2, but the transition amplitude is low [represented by the short magenta bar of the spectral function in (II)] and this leads to "low" current. (IV, V) On lowering the B-field further, the $|M_z = 2\rangle$ → $\big|\widetilde{M}_Z = -4\big\rangle$ transition is now allowed with the same relatively large change in M_Z (and higher nuclear polarization), again by a step of 2, but still with low transition amplitude [represented by the short cyan bar of the spectral function in (IV)]. Such a transition would merely lead to a continuation of the "low" current step (indicated by the thin dashed red line), and is more likely to occur if the B-field is swept slowly [we have enclosed cartoons depicting (IV, V) with a dashed line box to emphasis this condition]. (VI, VII) **Or** on lowering the B-field further and now with a higher nuclear spin relaxation rate (proportional to degree of nuclear polarization), the transition $|M_z = 1\rangle$ → $\big|\widetilde{M}_Z = -2\big\rangle$ transition will become more probable but now with a relatively small change in M_Z by a step of 1. Nonetheless, the transition amplitude is higher [represented by the taller red bar of the spectral function in

(IV)] so there is an "increment" in current. (VIII) The B-field is reduced further, **and in a more extended and realistic sequence of steps than shown**, the current grows "step-wise" as more probable transitions are sampled on moving through the spectral function tail on the low-energy side of the spectral function closer to its center. However, as the nuclear polarization builds up, nuclear spin relaxation becomes ever more significant. Eventually nuclear spin relaxation (just) outweighs further build-up of the nuclear spin polarization and the system can no longer compensate. (IX) When this happens allowed MW transitions are briefly centered at the maximum of the spectral function leading to a current "spike" but with vanishingly small change in M_Z. Thereafter, allowed transitions would essentially occur on the "wrong" (high-energy) side of the spectral function and transitions rapidly cease when the applied B-field becomes insufficient to maintain the effective Zeeman energy gap to sustain further MW transitions. At this point on the trailing edge of the EDSR signal, the current collapses, and any built-up nuclear polarization will relax naturally with time on a T_1 timescale (see Fig. 3 in main text).

Clearly the above description is highly simplified not least in the extremely limited number of steps considered and in the exaggerated height and width of the coarse "steps" in current. Also, we assumed the MW power is sufficiently low that only single spin-flip transitions are involved at each stage [see Fig. 2 (a) in main text and the discussion in Methods regarding Regimes I, II, III]. The interplay between a significant nuclear polarization being built-up and the increasing rate of nuclear spin relaxation as the nuclear spin builds up is not well represented. As demonstrated in the main text, we can attain pumped nuclear fields ~25 mT (~20% nuclear polarization) for slow (1 mT/min) sweep rates- see Fig. 2 (b) in main text. In line with expectations, Supplementary Fig. 4 shows that the increase in current (on the rising portion of the EDSR signal) as nuclear polarization builds up is more gradual for slow sweep rates than fast sweep rates, and this is also conducive for reaching larger nuclear fields. Lastly, a similar outcome would have been reached had we considered the case where the B-field is swept up from low to high field following the "mirror image" EDSR resonance (black trace in the current vs. B plot at the top of the Supplementary Fig. 12) other than the relevant transitions leading to nuclear polarization sampled on moving through the spectral function tail are now on the high-energy side of the spectral function. Our simple picture thus incorporates the bidirectional nature of dragging.

D. Microwave burst experiment and periodic oscillation of EDSR signal in wait time

We provide further commentary on the protocol implemented for the MW burst measurements shown in Fig. 4 of the main text. In particular we examine the role of each step in the sequence illustrated in the Fig. 4 (a) schematic of the main text and how the steps impact the proposed mechanism for the periodic oscillations in the EDSR signal. Supplementary Fig. 13 depicts two adjoining fixed period T=1 μs cycles of the Initialization-Manipulate-Wait-Read-Idle sequence of steps.

If the EDSR condition is met, during the Manipulate-step there is population transfer from the low-energy manifold hole state $|R\downarrow\rangle$ (hole ground state) to the high-energy manifold hole state $A|R\uparrow\rangle + b|L\downarrow\rangle$ (hole first-excited state: a mixed spin state) with the degree of transfer determined by the duration of the MW burst and the MW power. After the MW burst, the excited hole that is still (largely) trapped on the R QD can interact with the nuclear spin ensemble during the Wait-step. As discussed above, the SOI leads to finite x-component of the Knight field ($B_{Knight,x}$) which causes the nuclear spin quantization axis for the high-energy manifold to tilt away from the external B-field aligned along the z-axis. It is $B_{Knight,x}$ that is ultimately responsible for nuclear polarization. The hole spin state is interrogated during the Read-step when gates are pulsed temporarily to push the hole first-excited state energy level but not the hole ground state energy level above the Fermi energy in the drain contact. Successful readout empties the R QD, $B_{Knight,x}$ becomes zero, and the time evolution of the nuclear spin accumulated during the Wait-step is suspended. **The nuclear spin state that was precessing around the tilted nuclear spin quantization axis [in the bases …, $|\widetilde{M}_Z = -1\rangle$, $|\widetilde{M}_Z = 0\rangle$, $|\widetilde{M}_Z = +1\rangle$, …] is also simultaneously read out being projected against the bases …, $|M_Z = -1\rangle$, $|M_Z = 0\rangle$, $|M_Z = +1\rangle$, …].** Hole state $|R\downarrow\rangle$ is rapidly refilled and the trapped hole in the R QD is held ready during the subsequent Idle-step and Initialization-step for the next MW burst. The EDSR signal peak position in gate voltage as measured in the DC current (each data point corresponds to accumulated events over ~10^5 cycles) reflects any built-up nuclear polarization. In the measurements the initialization time, MW burst time, and read time are kept constant, and so as the wait time is varied the idle time is adjusted accordingly to maintain a 1 μs Initialization-Manipulate-Wait-Read-Idle cycle. We stress that the exit of essentially a spin-up hole in the excited state from the R QD to the drain during the Read-step followed by the fast reload of spin-down hole from the source to the R QD leads to a sudden switching of the effective magnetic field felt by the nuclei. Equivalently $B_{Knight,x}$ abruptly changes from being non-zero to zero and the tilt away from the z-axis of the nuclear quantization axis is lost.

How exactly do the holes that are pushed through the DQD over many cycles interact with the nuclear spins to drive nuclear polarization that is periodic in wait time? We consider the situation in Supplementary Fig. 14 which is grossly simplified but contains the essential physics. Crucially, the train of short MW bursts, as opposed to continuous MW modulation, makes it possible to form a superposition of nuclear spin states $\widetilde{M}_Z$. The number of nuclear spin basis states that can participate in the superposition is larger for shorter MW burst time.

Suppose initially we are in the state $|R\downarrow\rangle|M_z = 0\rangle$ and the detuning (changed here by adjustment of gate voltage rather than B-field) is such that MW transitions are possible. At the end of the first MW burst (during the *first cycle*), the time dependent state has become $|\Psi(t_{burst})\rangle = \alpha|R\downarrow\rangle|M_z = 0\rangle +$

$\beta|R\uparrow\rangle|\widetilde{M}_Z=-1\rangle+\gamma|R\uparrow\rangle|\widetilde{M}_Z=0\rangle$, i.e., there is probability the hole stays in the ground state with $|M_Z=0\rangle$, or is elevated to the hole first-excited state with $|\widetilde{M}_Z=0\rangle$ or $|\widetilde{M}_Z=-1\rangle$: assuming t_{burst} is of sufficient duration so that just these two states are accessible, i.e., $t_{burst}\sim 1/(E_{ZN}-|A_{||}|)$. During the Wait-step, the state evolves in time, and at the end of the Wait-step, the state is $|\Psi(t_{burst}+t_{wait})\rangle = \alpha|R\downarrow\rangle|M_z=0\rangle+\beta e^{i[E_0-(E_{ZN}-|A_{||}|)]t_{wait}}|R\uparrow\rangle|\widetilde{M}_Z=-1\rangle+\gamma e^{iE_0t_{wait}}|R\uparrow\rangle|\widetilde{M}_Z=0\rangle$ where we have set $\hbar=1$ for compactness, and where E_0, $E_{ZN}-|A_{||}|=g_N\mu_{B,N}B_z-|A_{||}|$, and $A_{||}$ are, as introduced is Section A above, the bare transition energy, the (reduced) Zeeman splitting in the high-energy manifold, and the Overhauser/Knight terms parallel to the external field respectively. During the Read-step, successful ejection of the hole to the drain, collapses the nuclear state and we must calculate the probability to find the nuclei in basis states, …, $|M_Z=-1\rangle$, $|M_Z=0\rangle$, $|M_Z=+1\rangle$, … (for the untilted quantization axis set by B_{ext}) rather than basis states …, $|\widetilde{M}_Z=-1\rangle$, $|\widetilde{M}_Z=0\rangle$, $|\widetilde{M}_Z=+1\rangle$, … (for the tilted quantization axis). At the moment the hole is ejected, the frozen nuclear state is $|\Psi(t_{end}=t_{burst}+t_{wait})\rangle = \beta e^{i[E_0-(E_{ZN}-|A_{||}|)]t_{wait}}|M_z\approx-1\rangle+\gamma e^{iE_0t_{wait}}|\widetilde{M}_Z=0\rangle = e^{iE_0t_{wait}}\left[\beta e^{-i(E_{ZN}-|A_{||}|)t_{wait}}|\widetilde{M}_Z=-1\rangle+\gamma|\widetilde{M}_Z=0\rangle\right]$. Eliminating the irrelevant phase factor, the nuclear state reads $|\Psi(t_{end})\rangle = \beta e^{-i(E_{ZN}-|A_{||}|)t_{wait}}|\widetilde{M}_Z=-1\rangle+\gamma|\widetilde{M}_Z=0\rangle$. We must now express the nuclear state carefully in terms of the …, $|M_Z=-1\rangle$, $|M_Z=0\rangle$, $|M_Z=+1\rangle$, … basis states. First-order perturbation analysis shows $|\widetilde{M}_Z=\Lambda\rangle=\varrho_\Lambda|M_Z=\Lambda-1\rangle+\sigma_\Lambda|M_Z=\Lambda\rangle+\tau_\Lambda|M_Z=\Lambda+1\rangle$, keeping the leading and first-order terms, and noting that generally $|\sigma_\Lambda|\gg|\varrho_\Lambda|=|\tau_\Lambda|$. For the frozen nuclear state, we find $|\Psi(t_{end})\rangle = \beta e^{-i(E_{ZN}-|A_{||}|)t_{wait}}(\varrho_{-1}|M_Z=-2\rangle+\sigma_{-1}|M_Z=-1\rangle+\tau_{-1}|M_Z=0\rangle)+\gamma(\varrho_0|M_Z=-1\rangle+\sigma_0|M_Z=0\rangle+\tau_0|M_Z=+1\rangle)$. To simplify the expression, without losing the essential physics, we set $\beta=\gamma=1$ and we take all ϱ_Λ values to be the same independent of Λ and so will drop the Λ index: hence forth we will use the symbol ϱ (and likewise for σ_Λ and τ_Λ we will use σ and τ). The expression now reads: $|\Psi(t_{end})\rangle = e^{-i(E_{ZN}-|A_{||}|)t_{wait}}(\varrho\,|M_Z=-2\rangle+\sigma\,|M_Z=-1\rangle+\tau\,|M_Z=0\rangle)+(\varrho_0|M_Z=-1\rangle+\sigma_0|M_Z=0\rangle+\tau_0|M_Z=+1\rangle)$. Dropping the two smaller terms in $|M_Z=-2\rangle$ and $|M_Z=+1\rangle$, and collecting the two larger (most significant) terms in $|M_Z=-1\rangle$ and $|M_Z=0\rangle$ the expression becomes: $|\Psi(t_{end})\rangle = (\sigma e^{-i(E_{ZN}-|A_{||}|)t_{wait}}+\varrho)\,|M_Z=-1\rangle+(\tau e^{-i(E_{ZN}-|A_{||}|)t_{wait}}+\sigma)\,|M_Z=0\rangle$. We find the probability that the nuclei are in state $|M_Z=-1\rangle$ is $P_{-1}=\sigma^2+\varrho^2+2\sigma\varrho\cos((E_{ZN}-|A_{||}|)t_{wait})$, and the probability that the nuclei are in state $|M_Z=0\rangle$ is $P_0=\sigma^2+\tau^2+2\sigma\tau\cos((E_{ZN}-|A_{||}|)t_{wait})$. Detailed analysis shows that if the sign of the terms in the Knight Hamiltonian product factor $A.b.A_\perp$ is positive, ϱ and σ have a positive sign and τ has a negative sign (we recall here that A and b reflect the influence SOI in the hole-first-excited-state manifold, and $A_\perp$ is the strength of the Overhauser/Knight field perpendicular to the

external field). Setting $\varrho = -\tau = \kappa$ where κ is strictly positive, $P_{-1} = \sigma^2 + \kappa^2 + 2\sigma\kappa\cos((E_{ZN} - |A_{||}|)t_{wait})$ and $P_0 = \sigma^2 + \kappa^2 - 2\sigma\kappa\cos((E_{ZN} - |A_{||}|)t_{wait})$. **Crucially we discover the phase factors in P_{-1} and P_0 are different.** If $(E_{ZN} - |A_{||}|)t_{wait} = 0, 2\pi, 4\pi, \ldots$ such that $\Delta t_{wait} = 2\pi/(E_{ZN} - |A_{||}|) = T_{As}$, where T_{As} is the period related to the inverse Larmor frequency for ^{75}As (we shall only consider one nuclear species), then P_{-1} has a maximum value and P_0 has a minimum value. If on the other hand, $(E_{ZN} - |A_{||}|)t_{wait} = \pi, 3\pi, 5\pi, \ldots,$ then P_{-1} has a minimum value and P_0 has a maximum value. The consequences are important. Dependent on t_{wait}, if for the *first cycle*, P_{-1} has a maximal value and P_0 has a minimal value, nuclear polarization is more likely, meaning also that for the (start of the) *second cycle*, the initial state $|R\downarrow\rangle|M_z = -1\rangle$ is favoured rather than $|R\downarrow\rangle|M_z = 0\rangle$. This means for $(E_{ZN} - |A_{||}|)t_{wait} = 0, 2\pi, 4\pi, \ldots,$ there is more chance to end up in $|R\downarrow\rangle|M_z = -2\rangle$ for *the second cycle*, i.e., promote the build up of nuclear polarization in this cycle (and likewise in subsequent cycles). If however, for the *first cycle*, P_0 has a maximal value and P_{-1} has a minimal value, nuclear polarization is less likely, meaning also that for the (start of the) *second cycle*, the initial state $|R\downarrow\rangle|M_z = 0\rangle$ is favoured rather than $|R\downarrow\rangle|M_z = -1\rangle$. This means for $(E_{ZN} - |A_{||}|)t_{wait} = \pi, 3\pi, 5\pi, \ldots,$, there is more chance to end up in $|R\downarrow\rangle|M_z = 0\rangle$ for *the second cycle*, i.e., suppress the build up of nuclear polarization in this cycle (and likewise in subsequent cycles).

Evidently, we have considered here a very limited number of cycles and MW transitions, and have only incorporated nuclear spin state terms $|M_Z = -1\rangle$ and $|M_Z = 0\rangle$. Over many more cycles, including yet more MW transitions, greatly expanding the number of nuclear spin terms visited, incorporating the slow change in detuning on sweeping a gate voltage (the DC current is measured every ~0.1 s reflecting events accumulated over ~10^5 cycles), and the competition between nuclear spin polarization and relaxation we would approach a more realistic situation encountered in the experiment. However, we assert, the oscillation of EDSR signal position with wait time reflects periodic reinforcement and suppression of nuclear polarization, corresponding to MAX and MIN positions of the EDSR signal in gate voltage respectively as sketched in Supplementary Fig. 14. We comment that in the worked example above, there is some arbitrariness in (absolute) phase depending on signs ϱ, σ, and, τ dependent on choice of sign of $A.b.A_{\perp}$. There is also some arbitrariness (variation) in the phase of the oscillation observed in the experimental data in Fig. 4 (d) of the main text probably related to the different nominal MW powers applied or to the delivered MW power varying with MW frequency if the nominal power level applied is the same. Experimentally we are principally interested in the period rather than the phase. Lastly, although we do not show the data here, a vertical section cutting through the EDSR resonance in Fig. 4 (b) at a MAX position reveals a line shape similar to that observed on sweeping the B-field down, e.g., see down-sweep trace in Fig. 1 (f), i.e., a gentle rise on the leading-edge culminating in a sharp peak before a sudden drop on the

trailing edge. Because of the SOI, the act of sweeping the B-field at constant detuning, or sweeping the detuning at constant B-field to alter the effective (GS-ES1) Zeeman energy gap is similar.

In summary, we have taken advantage of the SOI to periodically generate a finite x-component of the Knight field ($B_{Knight,x}$), and observe oscillations vs. wait time in the EDSR peak position commensurate with Larmor precession of the ^{75}As nuclei reflecting periodic build-up (reinforcement) of nuclear polarization or lack (suppression) of nuclear polarization.

References

[1] van der Wiel, W. G., De Franceschi, S., Elzerman, J. M., Fujisawa, T., Tarucha, S., & Kouwenhoven L. P. Electron transport through double quantum dots. *Rev. Mod. Phys.* **75**, 1 (2003).

[2] Hanson, R., Kouwenhoven, L. P., Petta, J. R., Tarucha, S. & Vandersypen, L. M. K. Spins in few-electron quantum dots. *Rev. Mod. Phys.* **79**, 1217 (2007).

[3] Studenikin, S. et al. Electrically tunable effective g-factor of a single hole in a lateral GaAs/AlGaAs quantum dot. *Commun. Phys.* **2**, 159 (2019).

[4] Studenikin, S. et al. Single-hole physics in GaAs/AlGaAs double quantum dot system with strong spin-orbit interaction. *Semicond. Sci. Technol.* **36**, 053001 (2021).

[5] Korkusinski, M. et al. (unpublished) (2025).

[6] Philippopoulos, P., Chesi, S. & Coish, W. A. First-principles hyperfine tensors for electrons and holes in GaAs and silicon. *Phys. Rev. B* **101**, 115302 (2020).

[7] See Mahan, G. D. Many-Particle Physics. (Kluwer Academic/ Plenum Publishers, 1981): For our situation, we take the spectral function to be $A(M_Z,\varepsilon) = \sum_{\widetilde{M}_Z} \left|\langle \widetilde{M}_Z | M_z \rangle\right|^2 \delta(E_{\widetilde{M}_Z} - E_{M_z} - \varepsilon)$, where M_Z $(\widetilde{M}_Z)$ is a nuclear spin basis state in the low-energy (high-energy) manifold with corresponding energy E_{M_z} ($E_{\widetilde{M}_Z}$), and ε is energy.

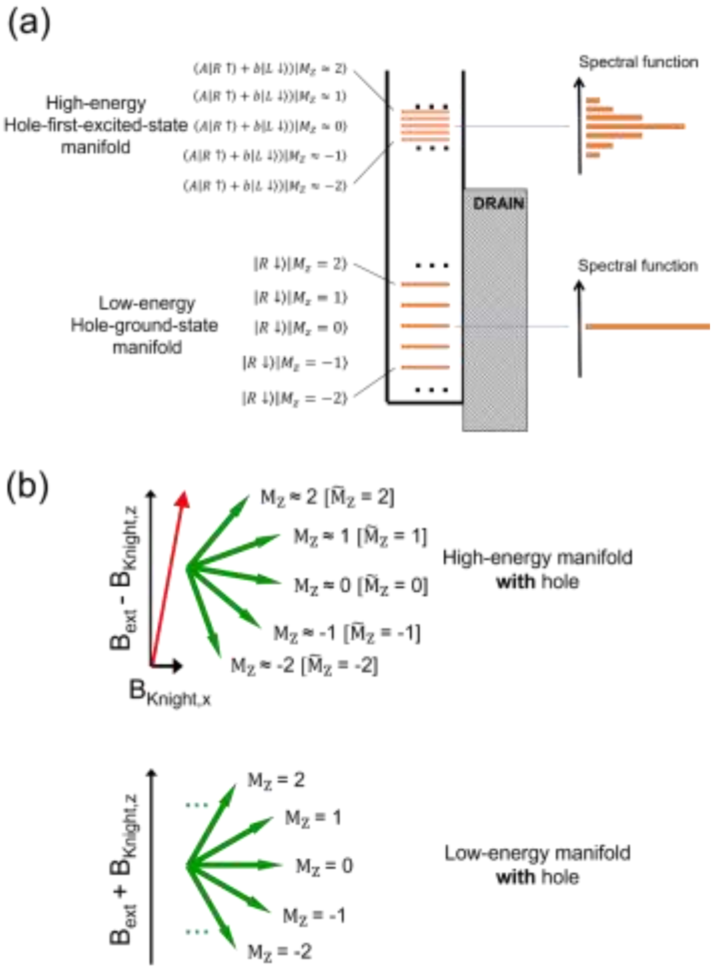

Supplementary Figure 11: (a) Sketch of molecular states in the hole-ground-state manifold and hole-first-excited-state manifold incorporating interactions ($A_{||}$ and $A_{\perp}$) between the hole and nuclear spins. The spectral functions keeping track of $|M_Z = 0\rangle$ state in both manifolds are also depicted. (b) Cartoons showing the orientation of the nuclear spin quantization axis relative to the external magnetic field B_{ext} for states in the low-energy manifold when occupied by a single hole and in the high-energy manifold when occupied by a single hole. The green arrows represent the relevant nuclear spin basis states. For illustration we have limited the number of nuclear spin basis states shown, and grossly exaggerated both the angle between neighboring nuclear spin basis states and the tilt of the total effective magnetic field induced by the finite x-component of the Knight field in the high-energy manifold.

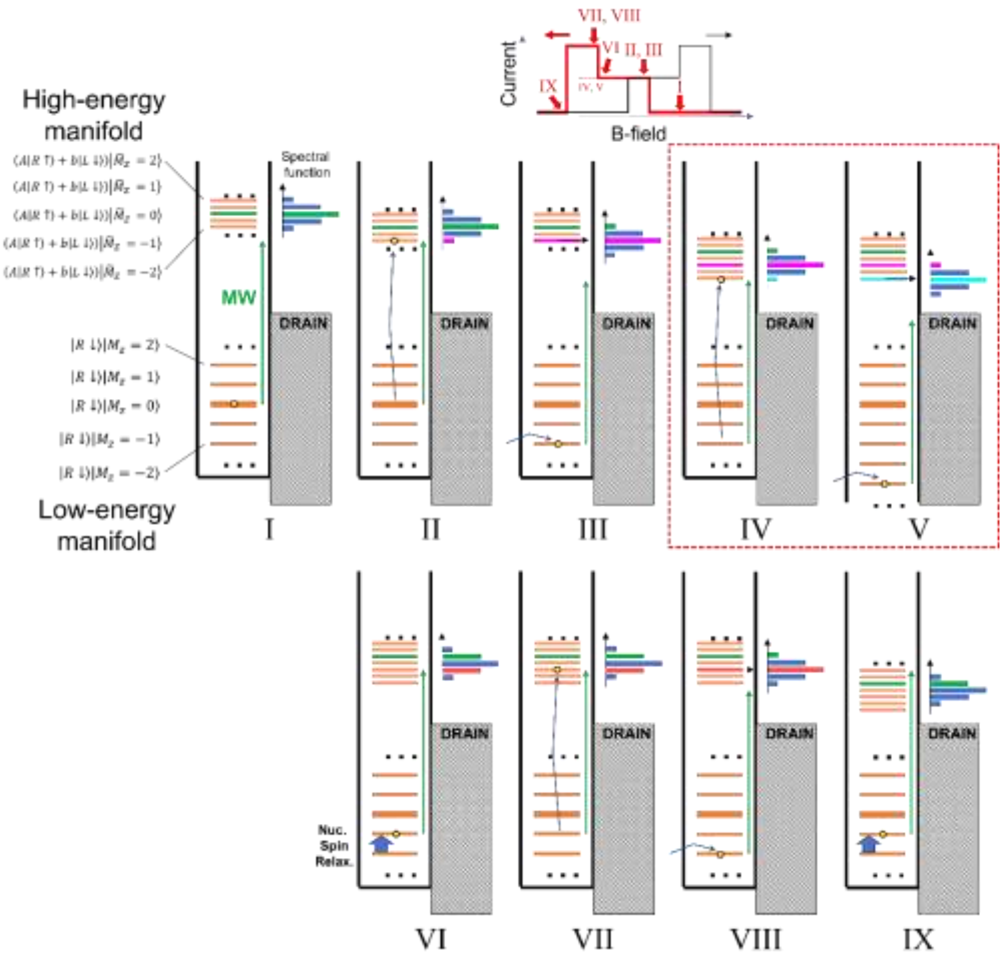

Supplementary Figure 12: Cartoon depicting elementary processes encountered during dragging and nuclear polarization in one simplified scenario. We consider only the scenario where the B-field is swept down from high to low field through the EDSR resonance indicated by the bold red trace in the current vs. B plot (top). Marked points I-IX on that trace correspond to the situations in the schematics below showing certain relevant low-energy and high-energy manifold basis states (essentially in the R QD), and transitions of holes (yellow circles) between these (strictly molecular-like) states under MW excitation, or the ejection of hole in an excited state to the drain or the reloading of a hole from the source (not shown) into the ground state. In panels I-IX, the fixed energy of the elementary allowed MW transition is indicated by the vertical green arrow labelled "MW", and as a reference level the $|R\downarrow\rangle|M_z = 0\rangle$ $((A|R\uparrow\rangle + b|L\downarrow\rangle)\big|\widetilde{M}_Z = 0\big\rangle)$ state energy level in the low-energy (high-energy) manifold is shown with a thick (green) line. Also shown in panels I-IX, are portrayals of the spectral function effectively indicating the amplitude of the transition into each level in the high-energy manifold from the indicated occupied hole level in the low-energy manifold. See text for discussion.

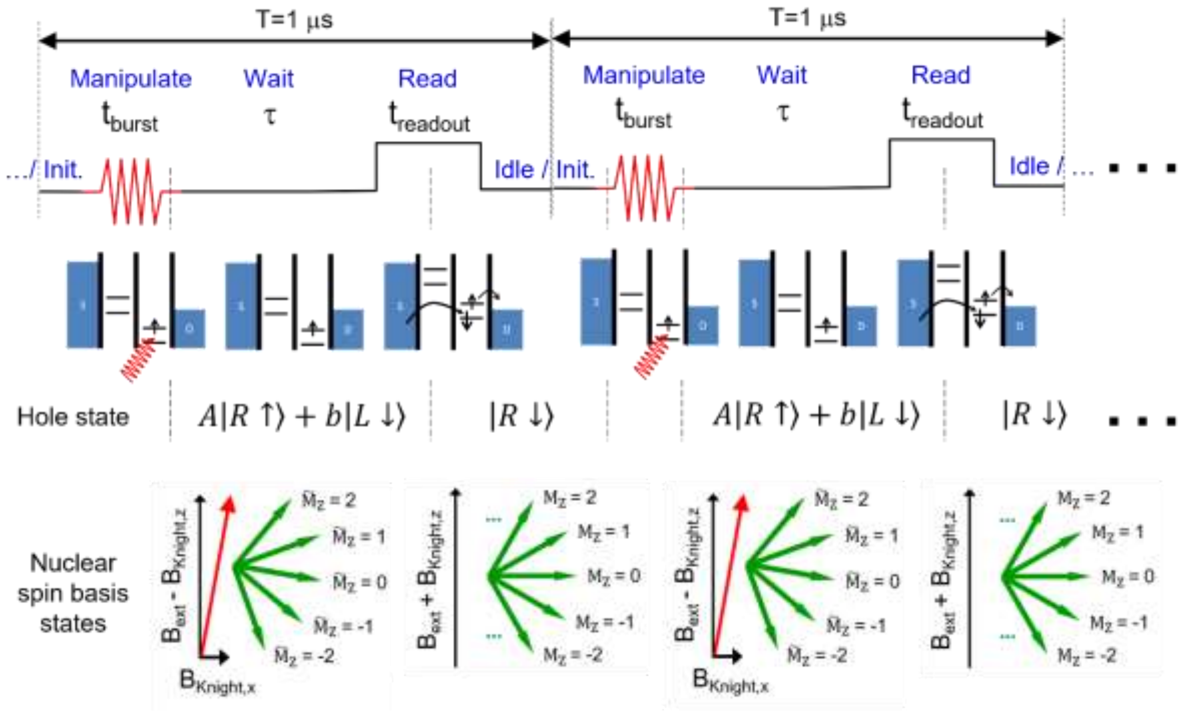

Supplementary Figure 13: Protocol for MW burst experiment under EDSR resonance condition. The steps in the Initialization-Manipulate-Wait-Read-Idle sequence for two 1 μs-period cycles are depicted along with the hole state and the quantization axis for nuclear spin states relative to the external magnetic field i. after the MW burst (variable length Wait-step) up to the point where the excited hole is ejected to the drain during the Read-step, and subsequently ii. from the point during the Read-step where a new hole is rapidly reloaded on to the R QD spin-down, through the subsequent (variable length) Idle-step into the (fixed length) Initialization-step up to the point where the next MW burst starts. The evolution with time of the hole state during the Manipulation-step, and the evolution with time of the nuclear spin state vector are not explicitly depicted here. The latter is examined in connection to Supplementary Fig. 14 which addresses the origin of the periodic oscillation of the EDSR signal.

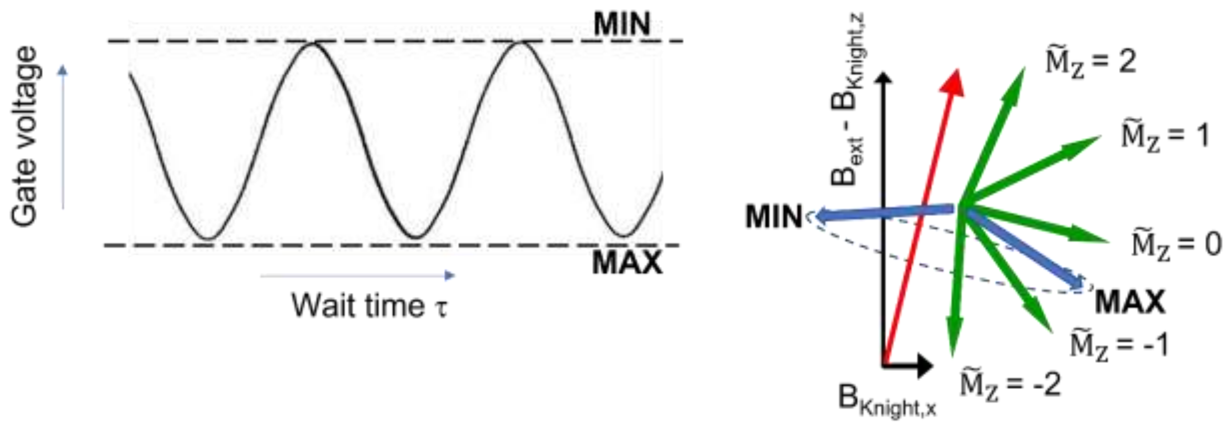

Supplementary Figure 14: Cartoon depicting origin of periodic oscillation of EDSR signal peak position in gate voltage as a function of wait time. The oscillations reflect the periodic evolution in the degree of nuclear spin pumping- the pumping is minimal (maximal) at the position indicated by MIN (MAX). Rotation of the nuclear spin state vector around the tilted nuclear spin axis occurs during the Wait step. When the excited hole exits to the drain during the Read step, the nuclear spin state vector has a small (large) projection on the z-axis at the position marked MIN (MAX) and the nuclear polarized is effectively reduced (magnified). We stress again for illustration we have limited the number of nuclear spin basis states shown, and grossly exaggerated both the angle between neighboring nuclear spin basis states and the tilt of the total effective magnetic field induced by the finite x-component of the Knight field in the high-energy manifold.
